# Residual hydrocarbons in long-term contaminated soils: implications to risk-based management

**DOI:** 10.1007/s11356-024-32593-7

**Published:** 2024-02-27

**Authors:** Md Mezbaul Bahar, Samarasinghe Vidane Arachchige Chamila Samarasinghe, Dawit Bekele, Ravi Naidu

**Affiliations:** 1 Global Centre for Environmental Remediation (GCER), College of Engineering, Science and Environment, University of Newcastle, Callaghan, NSW 2308 Australia; 2 crc for Contamination Assessment and Environmental Remediation (crcCARE), ATC Building, University Drive, Callaghan, NSW 2308 Australia; 3 Douglas Partners, West End, QLD 4101 Australia

**Keywords:** Petroleum hydrocarbons, Arid soils, Ecotoxicity, Natural attenuation

## Abstract

Petroleum hydrocarbon (PHC) contamination is a widespread and severe environmental issue affecting many countries’ resource sectors. PHCs are mixtures of hydrocarbon compounds with varying molar masses that naturally attenuate at different rates. Lighter fractions attenuate first, followed by medium-molar-mass constituents, while larger molecules remain for longer periods. This results in significant regulatory challenges concerning residual hydrocarbons in long-term contaminated soils. This study examined the potential risks associated with residual PHC and its implications for risk-based management of heavily contaminated soils (23,000–26,000 mg PHC/kg). Ecotoxicological properties, such as seedling emergence and growth of two native plant species—small Flinders grass (*Iseilema membranaceum*) and ruby saltbush (*Enchylaena tomentosa*)—and earthworm survival tests in PHC-contaminated soils, were assessed. Additionally, the effects of aging on the attenuation of PHC in contaminated soils were evaluated. Toxicity responses of plant growth parameters were determined as no-observed-effect concentrations: 75%–100% for seedling emergence, < 25%–75% for plant shoot height, and 75%–100% for earthworm survival. After 42 weeks of aging, the total PHC levels in weathered soils decreased by 14% to 30% and by 67% in diesel-spiked soil due to natural attenuation. Dehydrogenase enzyme activity in soils increased during the initial aging period. Furthermore, a clear shift of bacterial communities was observed in the soils following aging, including enrichment of PHC-resistant and -utilizing bacteria—for example, *Nocardia* sp. This study underscores the potential of natural attenuation for eco-friendly and cost-effective soil management, underlining that its success depends on site-specific factors like water content and nutrient availability. Therefore, we recommend detailed soil assessments to evaluate these conditions prior to adopting a risk-based management approach.

**Supplementary Information:**

The online version contains supplementary material available at 10.1007/s11356-024-32593-7.

## Introduction

Petroleum hydrocarbon (PHC) contamination is a widespread environmental concern impacting soil and groundwater in many regions around the world. PHC contamination in the terrestrial environment occurs through leakage from aboveground and underground storage tanks, spillage during transport of petroleum products, abandoned facilities, unplanned releases, and industrial processes. In Australia, mining has been a major part of the economy for a long time. While mining has enhanced the national economy by providing jobs for thousands of people, such operations have also led to soil and groundwater contamination. While remediation of PHC sites in urban environments has been achieved with much success, sites encountered in some rural and remote areas of Australia pose significant challenges given severe environmental conditions, including limited precipitation, the physicochemical properties of soils (for example, texture, pH, and mineralogy), and low nutrient and moisture content—factors that underpin natural attenuation.

Since PHC contains hazardous chemicals such as benzene, toluene, ethylbenzene, xylenes, and naphthalene, PHC contamination can endanger the health of plants, animals, and humans (Sarkar et al. 2005; Megharaj et al. 2011; Naidu et al. 2012). Thus, mineral industries in Australia are keen to ensure environmental sustainability by supporting research that helps remediate contaminated sites. While it has long been recognized that lighter volatile and semi-volatile hydrocarbon fractions in contaminated soils can be lost by volatilization, the compounds of residual heavier fractions (high molecular weight [HMW]) remain in soils. The remaining heavier and less soluble components are more likely to either sorb to soils or be present as free compounds coating soil colloid particles, particularly in soils that contain much organic material and fine particle sizes, and become less bioavailable (Clements et al. 2009). Toxicity information relating to the effects of PHC contamination on terrestrial ecosystems, particularly in PHC-containing soil that has been field weathered and is nutrient deficient, is limited.

“Aging” is a natural process of pollutants in the soil environment, affecting the bioavailability, biological toxicity, and biodegradability of pollutants. Aging or weathering is reported to diminish the toxicity of oil (Di Toro et al. 2007). Ecotoxicological assessment of contaminated soil could provide meaningful information in the characterization stage of ecological risk assessment. Ecotoxicity testing in combination with contaminant concentration measurements can be used as an efficient technique for assessing risks associated with contaminated, as well as remediated, soils in site assessments.

The use of biologically based endpoints may help appropriately define acceptable cleanup goals for weathered hydrocarbon-contaminated soils. Previous studies stated that the type of petroleum hydrocarbon and soil properties are essential in predicting the toxicological responses (Saterbak et al. 2000; Al-Mutairi et al. 2008). Ecotoxicity bioassays such as seed germination and plant growth have been used frequently to test the toxicity of contaminated soils. The plant species chosen for toxicity bioassays are generally easy-to-grow laboratory species that are occasionally sensitive to contaminants and soil properties. However, relatively few studies have used native wild plants for the ecotoxicological assessment of contaminated sites. The use of crops and vegetables for plant assay may not suit soils from arid/semi-arid regions. For such soils, using a native plant species may offer more accurate and reliable information on the toxicity of contaminants.

“Natural attenuation” refers to naturally occurring physical, chemical, and biological processes, or any combination of these processes, that reduce the mass, toxicity, mobility, volume, or concentration of contaminants in environmental media (Bekins et al. 2001). Natural attenuation is now commonly utilized to remediate groundwater contaminated with organic solutes, particularly PHCs and chlorinated solvents (Ling and Rifai 2007). Biodegradation by naturally occurring microorganisms is a major mechanism for the removal of PHC from contaminated soil. Although many case studies of the natural attenuation of PHCs in groundwater have been done, there are considerably fewer studies of natural attenuation of PHCs in soils. Historically, the remediation of soil contaminated with PHC has been expressed in terms of reductions in total PHC load rather than reductions in PHC risks to specified receptors. However, the risk-based management approach is based on demonstrating no or negligible, risks to humans and environmental receptors from contaminant exposure (Naidu et al. 2008). Thus, a thorough understanding of the potential risks of PHC-contaminated soils is needed prior to decisions on options for remediation. To this end, the primary objectives of this study were to investigate the ecotoxicological effects of HMW residual PHCs in long-term contaminated soils, including the kinetics of degradation under natural attenuation conditions. Based on these studies and findings, risk-based management of PHC-contaminated soils has been discussed.

## Materials and methods

### Soils and reagents

Soil samples that underwent weathering in the field were collected from PHC-effected locations from Newman, in the Pilbara region of Western Australia (WA). The climate of the Pilbara region ranges from semi-arid desert in the southeast to tropical on the coast. Contaminated soils from two locations with varying PHC levels (named as PHC soil A and PHC soil B) and non-contaminated control soil from a nearby site were transported to the laboratory using different intermediate bulk containers. Contaminated soils were dark in color, exhibited a strong petroleum odor, and contained stones and other debris (sticks, gravel, etc.). The soils were stored in a soil shed under ambient conditions for further processing and use.

### Soil characterization

PHC-contaminated and non-petroleum hydrocarbon contaminated (non-PHC) soil (as control) were characterized for their physicochemical parameters. The soils were air dried, homogenized, and sieved (2 mm mesh) before characterization. The samples’ pH and electrical conductivity (EC) were measured by using a pH meter in a soil suspension of 1:5 (w/v) soil:water (or 0·01 M CaCl_2_) after shaking for 1 h (Rayment and Higginson 1992). The dissolved organic carbon (DOC) content of the soil samples was determined in a 1:5 (w/v) soil:water slurry using a TOC analyzer (model TOC-L CSH, Shimadzu, China). Maximum water-holding capacity (WHC) was determined by flooding the soil in a cylinder fitted with a bottom filter and allowing it to drain for 48 h (Sarkar and Haldar 2005). Particle-size analysis was executed by two methods: (i) hydrometer and (ii) revised micropipette (Kettler et al. 2001). The soil moisture content was determined via the thermogravimetric method. Soil organic matter content was measured by the loss-on-ignition method (Moebius-Clune et al. 2017). Total nitrogen content was analyzed by the dry-combustion method using a carbon, nitrogen, and sulfur elemental analyzer (TruMac CNS analyzer, model 360–300-200, LECO Corporation, USA). The total metal concentrations of soils were investigated utilizing the US Environmental Protection Agency (EPA) Method 3051A: Microwave Assisted Acid Digestion of Sediments, Sludges, Soils, and Oils procedure (US EPA 2007).

Both total and water-soluble metals were extracted from the soil samples and assessed. For total metals, 0.5 g of air-dried soil was digested with 10 mL aqua regia in a programmable microwave digestion unit. The samples were then filtered and diluted with Milli-Q water. The metals Al, As, Ba, Ca, Cr, Fe, Mg, Mn, Mo, Na, and Zn were then measured using an inductively coupled plasma mass spectrometer (Agilent Technologies 7900 ICP-MS, model GB403A).

### PHC analysis

PHCs were grouped according to the National Environmental Protection Measures (NEPM) guidelines in Australia (Schedule B1, NEPM 2013a) and defined by four fractions. Fraction 1 (F1) ranges from nC_6_ to nC_10_, fraction 2 (F2) from > nC_10_ to nC_16_, fraction 3 (F3) from > nC_16_ to nC_34_, and fraction 4 (F4) from > nC_34_ to nC_40_ (NEPM 2013a). Total recoverable hydrocarbon (TRH) extraction was conducted according to the NEPM *Guideline on the Laboratory Analysis of Potentially Contaminated Soils* (Schedule B3, Method A2). Briefly, soils (5 g) were treated with anhydrous Na_2_SO_4_ and then extracted into 1:1 methylene chloride: acetone. The sample extract was then exchanged with hexane, and the TRH was analyzed by using a gas chromatography machine equipped with a flame ionization detector (Agilent Technologies, 7890B GC System, 7693 Autosampler). The results are reported as the amount of hydrocarbon in three different fractions: > nC_10_ to nC_16_, > nC_16_ to nC_34_, and > nC_34_ to nC_40_.

### Ecotoxicity tests

The soils used in the ecotoxicity testing were 100% control, 100% contaminated soils, and three dilutions of each of the PHC-contaminated soils. The dilutions of PHC-contaminated soils were prepared by mixing it with non-PHC (control) soil. For example, a 25% soil concentration was prepared with 25 weight percent (wt%) contaminated soil mixed with 75 wt% control soil. Dilutions were calculated on a dry-weight basis and were produced by hand mixing the soils. Due to the reduced organic matter content and limited nutrient availability in the control soil, artificial soil was employed as an alternative for toxicity evaluations. The artificial soil was prepared according to an Organisation for Economic Co-operation and Development (OECD) protocol (OECD 2006) and named “OECD soil” in this study. The OECD soil contains 70% sand, 20% clay, and 10% peat, and the pH was adjusted to 6.5 by adding CaCO_3_ to the mixture. Deionized water was added to moisten the soil before commencing the test.

The vegetation of Pilbara is characterized by grasslands, with grasslands and shrublands present in southern Pilbara. Seeds of 12 native plant species were procured from Nindethana Seed Service, Australia. The plants were screened by the germination viability of seeds in non-PHC (control) soil collected from Newman. Of the 12 species, seeds of only two plants germinated in the uncontaminated control soils, so these were selected for the toxicity testing of PHC-contaminated soils. The selected native plants were small Flinders grass (*Iseilema membranaceum*) and ruby saltbush (*Enchylaena tomentosa*).

Seedling emergence and growth tests were adapted from the OECD protocol (OECD 2006). For each test group, three replicates were performed. Small plastic pots were filled with OECD soil, non-PHC (control) soil, and PHC-contaminated soils with three dilutions of contaminated soil (25%, 50%, and 75%). Twenty small Flinders grass seeds and 20 ruby saltbush seeds were gently pushed into the soil surface in each pot. The pots were placed in plastic trays covered with perforated clear plastic wrap and kept in a temperature-controlled room (25 °C) in a 16 h light–8 h dark cycle. Seedling emergence was counted on days 3, 5, 7, and 14. When more than six small Flinders grass and four ruby saltbush seeds had germinated, the shoots of excess seeds were pinched off. The plants were harvested after 14 days of 50% emergence of the seedlings in the OECD and control soils. The plants’ shoot length, root length, and shoot biomass were then measured. The seedlings emerging after 1 week were not considered for the plant-growth assessment study.

The earthworm survival test was conducted according to OECD guidelines. Healthy earthworms were purchased from a local warehouse in Newcastle, Australia. Initial assessment showed that the earthworm does not survive in the field collected non-PHC and PHC-contaminated soils. Therefore, as for the ruby saltbush phytotoxicity study, both PHC-contaminated and non-PHC field soils were amended with 2% peat. The OECD soil was prepared as described in the previous section. Test containers used in this study were 500-L glass containers. OECD soil and different concentrations of PHC-contaminated soils (200 g) were put into glass jars in triplicate, and the soils were moistened with deionized water. Five adult earthworms were added to the soil, and the jars were closed with perforated lids. After transferring healthy earthworms to the test containers, their survival was monitored over a 14-day exposure period. The test was conducted in a controlled-temperature room (20 ± 2 °C, 16 h light–8 h dark cycle). The mortality of earthworms was assessed on the 7th and 14th days of incubation. The earthworms were not fed during the 2-week period, but moisture was added after 1 week to maintain the initial moisture level.

### Aging experiment design

The aging effect of natural attenuation of PHC-contaminated soils was investigated in a laboratory incubation study. Soils collected from Newman were hand homogenized and incubated at ambient conditions. To determine the aging effect of a recently contaminated soil, non-contaminated soil was spiked with diesel at similar concentration to that of field-contaminated soil A. The average TRH concentrations in the homogenized field-contaminated soil A and soil B were 21,900 ± 990 mg/kg and 26,000 ± 670 mg/kg, respectively, and 23,000 ± 150 mg/kg in the diesel-spiked control soil. Deionized water was added to achieve a moisture content of approximately 40% and 60% of the WHC of the soils. Hence, two moisture levels—that is, low moisture (40% of WHC) and high moisture (60% of WHC)—were maintained in all soils with three replicates.

The soils were incubated in glass jars with perforated lids in the shed house laboratory located at the Callaghan campus, University of Newcastle. Diurnal variations of temperature and relative humidity were recorded during the incubation period (Fig. S1). Soils were sampled at 0, 2, 6, 10, 18, 26, 34, and 42 weeks for chemical and microbiological analyses. Moisture content was measured during the sampling, and when the moisture level had dropped below target level, the soils were moistened back to the target level (Fig. S2).

### Soil enzyme activity and microbial diversity analysis

The dehydrogenase activity (DHA) was determined by the reduction of triphenyltetrazolium chloride (TTC) to triphenylformazan (TPF) (Tan et al. 2017). Approximately 3 g of soil with field moisture was added into a 50-mL centrifuge tube, followed by adding 0.5 mL TTC aqueous solution, 30 mg CaCO_3_, and 1.25 mL of Milli-Q water. After being mixed thoroughly, the tubes were statically incubated at a constant temperature (37 °C) in the walk-in room for 24 h. Then 10 mL methanol was added, and all tubes were shaken vigorously before being centrifuged at 4000 rpm for 15 min. The supernatant was obtained and analyzed using a spectrophotometer (Model BIOMATE 3S, Thermofisher Scientific) at 485 nm. All subsequent results were expressed based on the dry weight of soil in µg TPF g^−1^ h^−1^.

Microbial diversity profiling is conducted to identify the relative proportion of microorganisms present in a mixed microbial community. Samples from diesel-spiked soil and two PHC-contaminated soils were collected during week 0 and week 34 of the aging-incubation study and the microbial profiling was performed using the Illumina MiSeq platform at the Australian Genome Research Facility (AGRF), Melbourne, Australia. The hypervariable V3–V4 region of the 16S rRNA gene was amplified using a 341F-806R primer set for the bacterial community. However, based on the initial polymerase chain reaction (PCR) and indexing fluorometry of the samples, samples from PHC soil A did not meet the minimum quality control (QC) requirement to generate sequencing output. Therefore, the samples from diesel-spiked soil and PHC soil B were selected to be sequenced for bacterial diversity analysis.

### PHC degradation kinetics

Kinetic analyses were carried out to estimate the rates of PHC biodegradation in each microcosm treatment studied. The first-order point decay rate constant (*k*_point_) for different fractions of TRH was calculated by plotting the concentration of TRH fractions versus aging-incubation time of the contaminated soils. The time required to reach a remediation goal (NEPM ecological screening levels [ESLs]) was calculated from the first-order exponential decay kinetics for TRH fractions F2–F4 using (Newell 2002):

1 $${C}_{t}={C}_{0}{e}^{-kt}$$

where *C*_*t*_ is the concentration at time *t*, *C*_*0*_ stands for initial concentration at time 0, and *k* denotes the first-order decay rate constant.

The first-order decay rate (*k*) is used in the calculation of the estimated time to clean up the weathered soil and diesel-spiked soil (CDLE 2002). Rearranging the first-order decay equation (Eq. 1) yields

2 $${C_G/C}_0=e^{-kt}\;\mathrm{and}\;{t=\lbrack-\text{ln}(C}_G{/C}_0)\rbrack/k$$

where ${C}_{G}$ is the concentration of the cleanup goal (NEPM ESLs) (mg/kg), ${C}_{0}$ represents the current concentration of TRH fractions in soil (mg/kg), $k$ is the decay rate (per year), and $t$ is the time for ${C}_{0}$ to attenuate to ${C}_{G}$ or NEPM ESLs (years).

### Statistical analysis

The no-observed-effect concentration (NOEC) levels are useful when assessing the risk of contaminated sites. For seedling emergence, shoot/root length, shoot biomass, and earthworm survival, NOECs were obtained by statistically comparing the responses in the field PHC-contaminated soils with those in the control soils. Dunnett’s test for multiple comparisons was used to compare each treatment mean to the control soil’s mean. The analyses were performed at an *α* of 0.05 using SPSS Statistics software (version 25, IBM, USA). The calculations of mean and standard deviation of the results were conducted using an Excel (Microsoft Office 2016) spreadsheet, while the nonlinear regression analysis was undertaken with SPSS.

## Results and discussion

### Soil properties

The baseline physicochemical properties of the three soil samples used in this study are listed in Table 1. The pH_water_ of the soils ranged from 7.3 to 8.0, indicating the soils were neutral to slightly alkaline, which is considered optimum for microbial activity and biodegradation of PHC in soils (US EPA 2017; Koolivand et al. 2022). EC in the control soil (2167 µS/cm) was higher than in the PHC-contaminated soils (1591–1701 µS/cm). The cation exchange capacity of the soils ranged from 3.46 to 3.93 cmol( +)/kg. The soil organic matter varied from 1.1% to 5.4%. The total nitrogen content in the soils ranged from 180 to 465 mg/kg, and total phosphorus ranged from 161 to 559 mg/kg. Nitrate–N content in the contaminated soils was very low (0.5 mg/kg), and no ammonium-N was detected in any of the soils.

**Table 1:** Physicochemical characteristics of soils from Newman, Western Australia

Soil property	Control soil	PHC soil A	PHC soil B
pH			
In H_2_O	7.99 ± 0.1	7.44 ± 0.1	7.56 ± 0.1
In 0·01 M CaCl_2_	7.86 ± 0.1	7.30 ± 0.1	7.43 ± 0.1
EC (µS/cm)	2167 ± 110	1591 ± 43	1701 ± 24
CEC_B_ (cmol( +)/kg)	3.58 ± 2.9	3.93 ± 0.1	3.46 ± 0.2
Water-holding capacity (%)	18.5 ± 0.2	23.9 ± 0.3	23.8 ± 0.4
Texture			
Sand (%)	56	62	58
Silt (%)	33	29	31
Clay (%)	11	9	11
Soil organic matter (%)	1.1 ± 0.3	5.4 ± 0.8	5.3 ± 0.8
Total N (mg/kg soil)	465 ± 92	180 ± 28	220 ± 30
Total P (mg/kg soil)	559 ± 21	231 ± 30	161 ± 171
Nitrate–N (mg/kg soil)	97.4 ± 5.1	0.4 ± 0.1	0.5 ± 0.1
Ammonium-N (mg/kg soil)	Not detected	Not detected	Not detected

*CEC*_*B*_, cation exchange capacity; *EC*, electrical conductivity

The concentrations of metals in the control soil and PHC-contaminated soils differed, and most the metals of potential concern did not exceed the ecological investigation levels (EILs) set by the WA Department of Environment and Conservation (2010), except for Mn and Mo (Table 2). The concentration of Mn in non-PHC (control) soil was two-fold higher than in PHC-contaminated soils. However, the water-soluble Mn content was higher in the PHC-contaminated soils than in the control soil (Table 2). The concentration of total Sr in the control soil was 11 mg/L, twice that in the PHC-contaminated soils. Similarly, the water-soluble Sr in the control soil was two-fold higher than in the PHC-contaminated soils. The water-soluble fraction of Sr was higher than that of other trace metals except Mn in all soils. Although Sr can be released from the weathering of sulfate minerals and ion-exchange reactions with clay minerals (Siegel and Bryan 2014), aeolian dust input can be a major Sr source in arid regions (Coble et al. 2015). The Mo content in the control soil was less than the EILs (40 mg/kg) but nearly 10 times higher than the EILs in the PHC-contaminated soils. Mo is extensively utilized in the petroleum industries as a catalyst, particularly for sulfur removal from crude petroleum (Lennartson 2014). Consequently, this leads to an anticipated elevated concentration of Mo at PHC-contaminated sites. Given the significance of Mo in soil health and its potential toxicity at elevated levels, understanding its origin is crucial for targeted remediation efforts. Potential remediation approaches of Mo include phytoremediation and soil washing methods to lower Mo levels. Additionally, adjusting soil pH can influence Mo availability and mobility, potentially aiding in its management.

**Table 2:** Total metal content in soils. The numbers within parentheses indicate mean water-soluble concentration

Metal	Concentration of metals (mg/kg)			
	Control	PHC soil A	PHC soil B	Ecological investigation level (DEC WA 2010)
Al	8555 ± 341	8714 ± 840	8081 ± 1633	
As	11 ± 1	9 ± 0	10 ± 0	20
Ba	34 ± 1 (0.17)	43 ± 1 (0.61)	42 ± 3 (0.52)	300
Ca	3426 ± 266	2241 ± 1875	1569 ± 463	
Cr	56 ± 3	82 ± 3	78 ± 1	400
Fe	179,436 ± 2889	121,094 ± 7836	125,869 ± 8062	
Mg	4684 ± 79	4031 ± 211	4030 ± 523	
Mn	1120 ± 80 (0.02)	539 ± 39 (17.32)	525 ± 61 (14.77)	500
Mo	10 ± 3 (0.2)	349 ± 28 (0.23)	398 ± 161 (0.2)	40
Na	551 ± 100	451 ± 173	555 ± 443	
Sr	11 ± 0.3 (1.94)	4.6 ± 0.8 (0.81)	4.3 ± 0.5 (0.84)	
Zn	91 ± 4	136 ± 12 (0.32)	151 ± 13 (0.27)	360

*DEC, WA*: Department of Environment and Conservation, Western Australia; *PHC*: petroleum hydrocarbon

**Fig. 1:**
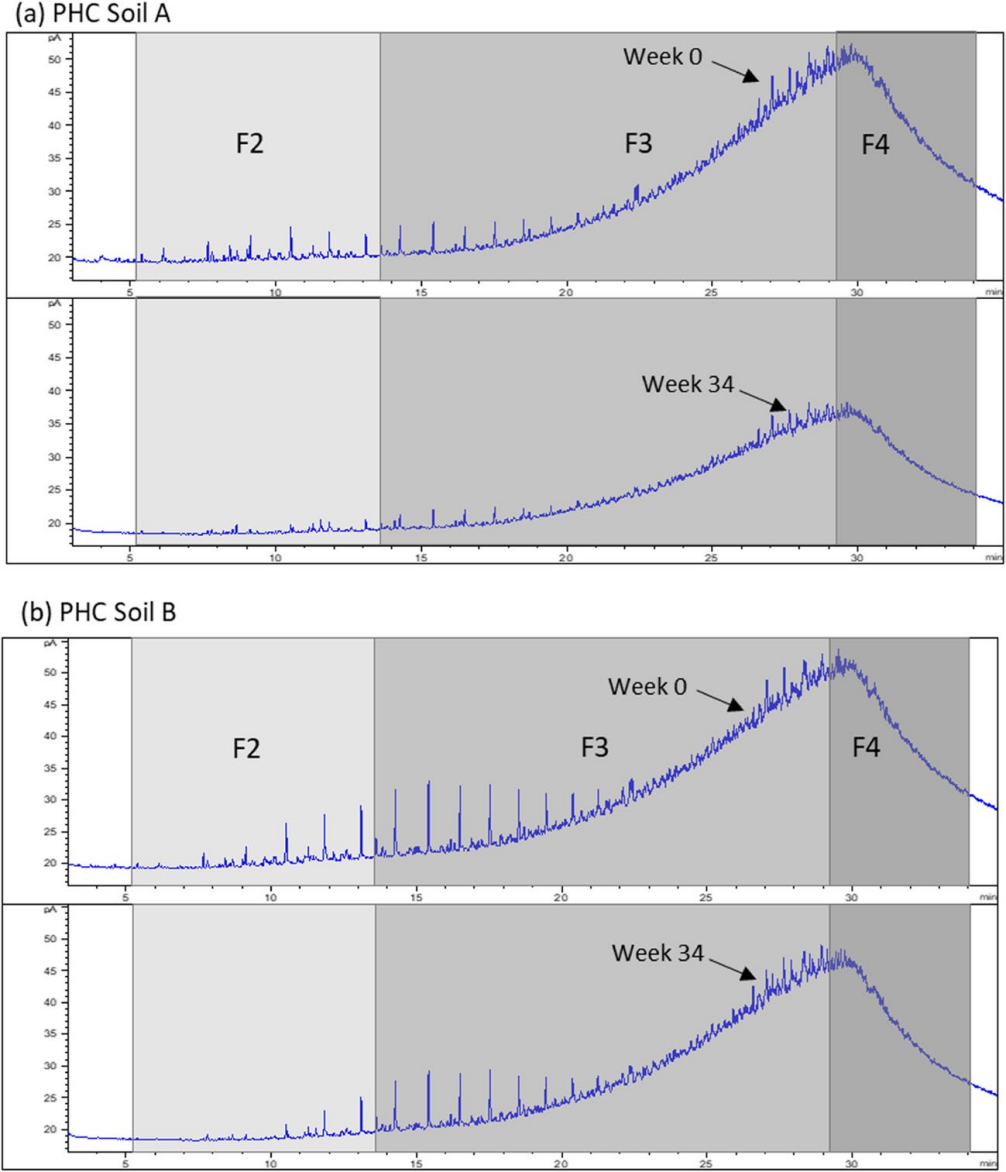
Gas chromatography with flame ionization detection chromatograms of petroleum hydrocarbons (PHCs) in contaminated soils measured at the beginning of the aging incubation and after 34 weeks: (**a**) PHC soil A and (**b**) PHC soil B. F2, F3, and F4 represent different TRH fractions, which are separated based on their retention times as indicated by light to dark gray shading

TRH (> nC_10_ to nC_40_) concentration in contaminated soil A was 21,900 ± 900 and 26,000 ± 670 mg/kg in soil B. Both values indicate that the two soils contained elevated PHC concentrations. The concentrations of TRH F1 (nC_6_ to nC_10_) in all three were below the limit of reporting; that is, < 10 mg/kg. Other TRH fractions—F2 (> nC_10_ to nC_16_), F3 (> nC_16_ to nC_34_), and F4 (> nC_34_ to nC_40_)—in the contaminated soils, were found to be higher than the NEPM ESLs. The NEPM ESLs for different TRH fractions and land uses are described in the *National Environment Protection (Assessment of Site Contamination) Measure 1999* (ASC NEPM) (NEPC 2006), Schedule B1 (NEPM 2013a). A qualitative assessment of TRH can be determined using trace gas chromatograms. The “hump” of hydrocarbons, which is also represented by unresolved complex mixture, indicates that the TRH in both soils is moderately to heavily weathered (Fig. 1).

### Ecotoxicological assessment of PHC soils

Seedling emergence and growth tests were conducted for the two native plants in different dilutions of the field PHC-contaminated soils. The seedling-emergence rate of both the plants was generally higher in OECD artificial soils than in the control soil. Emergence of small Flinders grass (*I. membranaceum*) seedlings in all the soils started early, but some emerged after 2 weeks (Fig. S3). After 1 week, the emergence of small Flinders grass seedlings in OECD soil was 47%, while in the control soil and PHC soil A, it was 18% and 12%, respectively. No significant difference (*p* ≤ 0.05) in seedling emergence was observed between the control soil and different dilutions of PHC soil A. In the case of PHC soil B, there was no significant difference between the control soil and 50% of PHC soil B, but the difference was marked with 75% and 100% of PHC soil B. The initial measured TRH (> nC_10_ to nC_40_) concentrations in soil A and soil B were 21,900 ± 990 mg/kg and 26,000 ± 670 mg/kg, respectively.

Initial assessment of ruby saltbush (*E. tomentosa*) seed germination showed that the seedling emergence was < 10% in in both field PHC-contaminated soil and non-contaminated soil (data not shown). The germination rate could have been affected by the minimal organic matter content in arid soils and hence low moisture-holding capacity of soils. Soil organic matter has a considerable influence in increasing water retention in soil (Lal 2020). Therefore, the field soils were amended with 2% peat, which helps maintain soil moisture. These amended soils were then used for the toxicity assay with ruby saltbush. A preliminary trial was conducted using different proportions of peat to field soils to determine the minimum amount of peat to be added in the field soils, the objective being to preserve the initial characteristics of the field soils. The peat moss was air dried and sieved (< 2 mm) before mixing with the soils. Then the soils were prepared for the seedling-emergence test as described in the “Ecotoxicity tests” section. After 1 week, the emergence of ruby saltbush seedlings in OECD soil was 69%. Adding peat improved the soil properties, and, as a result, seedling emergence of 50% was observed in the peat-amended control soil (Fig. S4). After amending with peat, 25% of ruby saltbush seedlings emerged in the PHC soil A 100% group. However, even after mixing with peat, no seedling emerged in the PHC-contaminated soil B 100% group.

The average shoot/root lengths and shoot biomass of small Flinders grass seedlings grown in OECD soils were twice those of the seedlings grown in control soil (Fig. S5). The shoot/root length declined with the increasing concentration of PHC soil A and PHC soil B. No seedlings survived in the 100% PHC-contaminated soil B. The shoot-biomass results of small Flinders grass in all the treatment soils were similar to the shoot-length results. Like seedling-emergence study, the growth study of ruby saltbush seedlings was conducted in peat-amended (2%) field soils. The shoot length of ruby saltbush seedlings in OECD soil was less than that of those in control soil, whereas the root length in these soils was similar (Fig. S6). Both the shoot and root length reduced with the increasing concentration of PHC soil A and B. The shoot biomass of ruby saltbush in all treatment soils revealed results similar to the shoot-length results.

An earthworm mortality test was conducted in the soils similarly to the phytotoxicity tests but with different growth conditions. After 14 days of exposure, > 90% of earthworms survived in all control soils (OECD soil and peat-amended control soil) (Fig. S7). The initial toxicity seen in the PHC-contaminated soils (during initial assessment without peat) was considerably reduced following amendment with 2% peat. However, the responses of earthworm survival varied in the PHC-contaminated soils. In PHC-contaminated soil A, the survival rate was > 80% in the soils containing up to 75% of the PHC in soil, whereas in PHC soil B, > 80% survival was recorded in soils with up to 50% of the PHC in soil. The lowest survival was recorded in 100% PHC soil A. These results confirm that the peat amendment improves the soil quality for the survival of earthworms. The ecological safety of the PHC-effected soils in these sites can be recovered by amending the soils with an appropriate amount of peat.

Commonly, NOEC levels are used in the risk assessment of contaminated sites. For seedling emergence, shoot/root length, shoot biomass, and earthworm survival, NOECs were obtained by statistically comparing the responses in the treatment soils with those in the control soils. In terms of the emergence of small Flinders grass seedlings, the NOECs of the PHC-contaminated soils A and B were 100% and 75%, respectively, concentration of these soils (Table 3). Ruby saltbush was more sensitive than small Flinders grass. The toxicity of the PHC soils was high (lower NOECs) when the seedling growth parameters were considered. NOEC values for the earthworm survival endpoint were 75% and 100% concentration of contaminated soil A and soil B, respectively (Table 4). The low toxicity of contaminated soils to earthworms could be due to peat amendment prior to the test. Overall, the soils were toxic to the ecological receptors tested in this study and require a cleanup to meet the regulatory guideline. For these reasons, we recommend native plants for assessing the risk of soils without organic amendments. To bridge the gap between ecotoxicological data and risk-based soil management, it is suggested that the findings from this study can be incorporated into a risk assessment framework. Such a framework can evaluate the ecological risk associated with residual PHC by considering the direct toxicity to key soil organisms as well as the overarching effects on soil health and ecosystem services. Studies on the ecotoxicological responses of plants and earthworms provide critical baseline data for this framework, highlighting the sensitivity of local biota to PHC contamination and identifying threshold levels that could guide remediation targets.

**Table 3:** Native plant growth test—results calculated as no-observed effect concentrations (NOECs)— for field PHC-contaminated soils

Native plant	Toxicity test parameter	NOEC (percent concentration of contaminated soil)	
		PHC soil A (TRH sum: 21,900 mg/kg)	PHC soil B (TRH sum: 26,000 mg/kg)
Small Flinders grass (*Iseilema membranaceum*)	7-day germination	100	75
	14-day germination	100	75
	Shoot length	75	25
	Root length	50	< 25
	Shoot weight	50	25
Ruby saltbush* (*Enchylaena tomentosa*)	7-day germination	50	< 25
	14-day germination	50	< 25
	Shoot length	< 25	25
	Root length	75	25
	Shoot weight	< 25	< 25

*The soils were amended with 2% peat for the ruby saltbush plant-growth test

*PHC*, petroleum hydrocarbon; *TRH*, total recoverable hydrocarbon

**Table 4:** Earthworm survival test —results calculated as no-observed effect concentrations (NOECs)— for field PHC-contaminated soils

	NOEC (% concentration of contaminated soil)	
Earthworm test*	PHC soil A (TRH sum: 21,900 mg/kg)	PHC soil B (TRH sum: 26,000 mg/kg)
7-day survival	75	100
14-day survival	75	75

*The soils were amended with 2% peat for the earthworm survival test

*PHC*, petroleum hydrocarbon; *TRH*, total recoverable hydrocarbon

### PHC attenuation

**Fig. 2:**
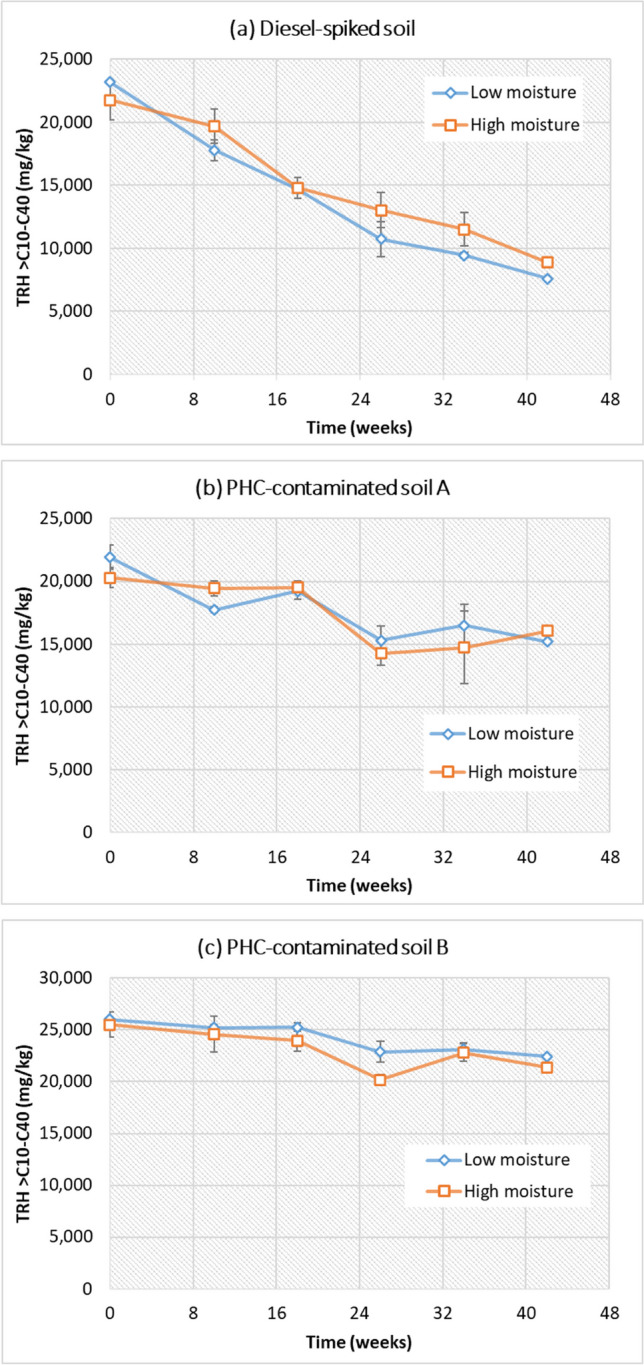
Total recoverable hydrocarbon (TRH) > nC_10_ to nC_40_ in soils during the aging-incubation period

TRH value for fractions F2–F4 was determined according to the NEPM *Guideline on the Laboratory Analysis of Potentially Contaminated Soils* (Schedule B3, NEPM 2013b, Australia). The preliminary soil characterization showed that the TRH F1 was negligible and consequently could be ignored in this study. So, the TRH in this study represents the sum of F2–F4. The concentrations of TRH (sum: > nC10 to nC40) in diesel-spiked and field-contaminated soils at different time intervals are presented in Fig. 2. The initial TRH concentrations in the diesel-spiked soil, PHC-contaminated soil A, and PHC soil B were 23,000 mg/kg, 22,000 mg/kg, and 26,000 mg/kg, respectively. TRH content in each soil waned over 42 weeks’ aging incubation, and there was no significant difference at two soil moisture conditions (as indicated by 40% and 60% of WHC). Degradation of TRH occurred more quickly in the diesel-spiked soil than in the field-weathered soils. After 42 weeks’ natural attenuation under low moisture conditions, TRH in diesel-spiked soil decreased from 23,000 to 7,600 mg/kg. This represents a TRH removal efficiency of 67%. In contrast, the TRH removal in PHC-contaminated weathered soils was 14%–30%. Although other natural processes such as volatilization can contribute to a portion of the PHC reduction, Margesin and Schinner (1997) reported that the abiotic loss of diesel fuel in the first 30 days was less than 10% at 25 °C.

Bioremediation has been considered an effective method for degrading PHC in weathered PHC-contaminated soil. In the aging experiment of this study, PHC degradation seemed to be limited in the heavily weathered soil (PHC soil B). This could be because of the low bioavailability of aged hydrocarbon residues to the metabolically competent degrading microorganism. Also, the weathered soil contains the mostly heavier fraction of hydrocarbon compounds that would be difficult to break down to daughter compounds or completely mineralize.

### Soil enzyme activities in soils

The enzymatic activity of the soil and proliferation of soil microorganisms are the best indicators of a soil ecosystem’s stability and fertility. This is due to the immediate response of the biochemical activity of the soil to any disturbance in the environment. Among all enzyme groups of the soil environment, dehydrogenases belong to one of the most important enzymes because they occur in all living microorganism cells (Kaczyńska et al. 2015). Since they are closely connected with microbiological redox processes, dehydrogenases are often considered an index of the general microbiological activity of the soil.

The DHA was determined by reduction of TTC to TPF, as described in the “Soil enzyme activity and microbial diversity analysis” section. The initial DHA values in the control soil and two field-contaminated soils (PHC soil A and PHC soil B) were 0.052, 0.280, and 0.255 µg TPF g^−1^ h^−1^, respectively. The addition of moisture stimulated the DHA in all the soils during the aging process (Fig. S8). The DHA was lower in the high moisture conditions (60% of soil WHC) than in the low moisture conditions (40% WHC). Soil moisture level has been shown to demonstrate a positive effect on DHA (Frymark-Szymkowiak and Karliński 2022). However, when the moisture ranges reach saturation, the increased moisture may inhibit the aerobic activities in soil (Tate and Terry 1980). Spiking the control soil with fresh diesel significantly increased the DHA until week 26, when it fell. Similarly, in the field-contaminated weathered soil, the DHA increased until week 10 and then started decreasing and remained constant. The DHA in the control soil also reduced after week 10, and virtually no activity was measured after 34 weeks’ incubation. The higher DHA in diesel-spiked soils reflects enhanced microbial activity and higher PHC degradation.

Being intracellular enzymes, dehydrogenases are often mentioned as a good indicator for estimating contaminants, primarily PHCs. The degradation rate of hydrocarbons in oil products generally increases when more DHA is directly involved in this process (Kaczyńska et al. 2015). The high activity of the dehydrogenase enzyme in the diesel-spiked soils have may resulted from the intensified growth of microorganisms that use diesel as their carbon source. However, microbes also require other essential nutrients such as nitrogen and phosphorus for metabolism and growth. In natural attenuation, no biostimulating nutrients are added. This results in limiting the essential nutrients in the soil, thereby slowing down the microbial activity and degradation of hydrocarbon compounds.

### Bacterial community shift

Soil microbial activities and community structures wield a profound influence on the degradation of PHC. Studies have shown that the bacterial community is mostly responsible for degrading both aliphatic and aromatic hydrocarbon compounds (Varjani 2017; Haritash and Kaushik 2009; Das and Chandran 2011; Megharaj et al. 2011). The biodegradation of PHCs by indigenous bacterial communities has been well documented. Therefore, the shift of the bacterial community and relative abundance of any particular group can be a good indicator for evaluating the petroleum contamination and remediation success.

**Fig. 3:**
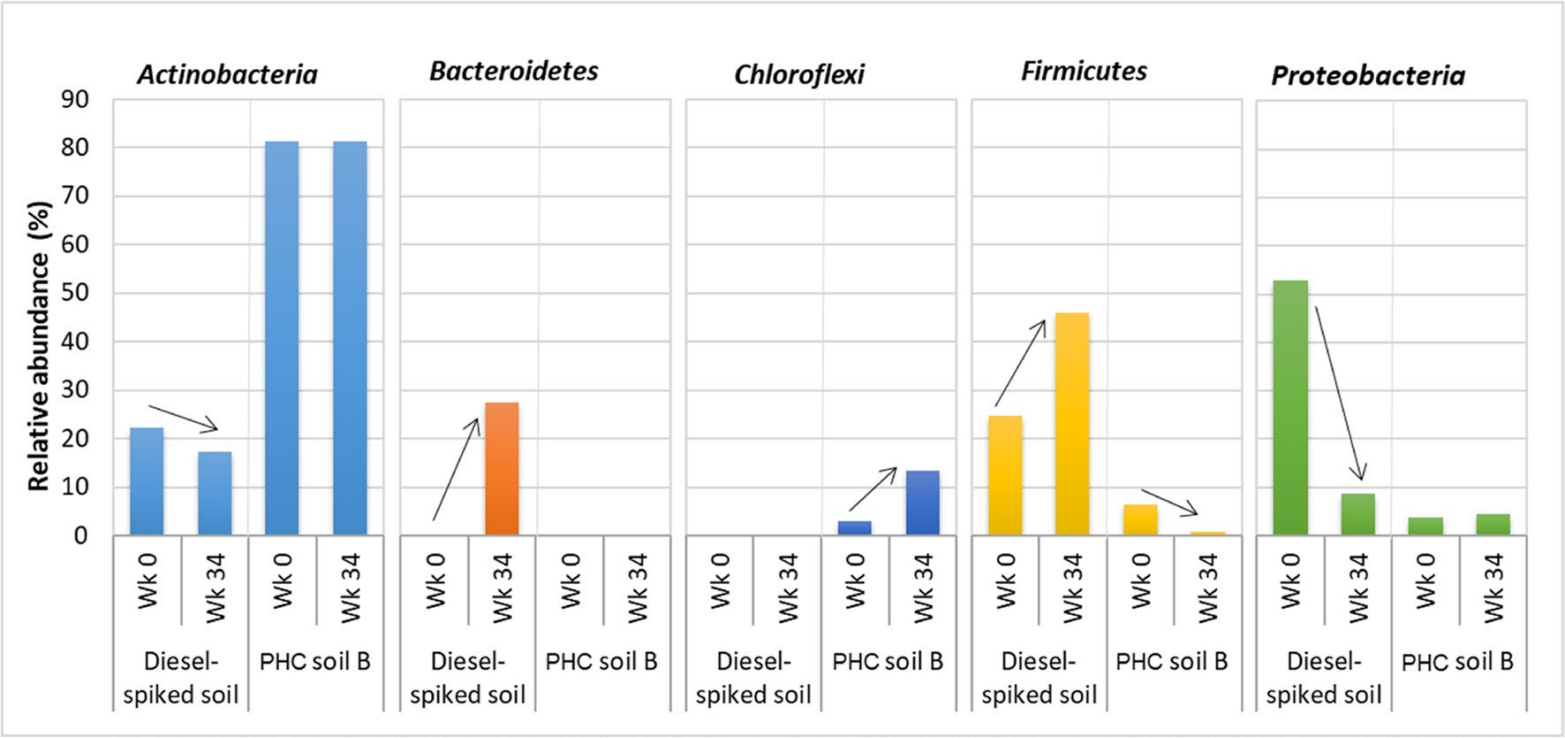
Changes of the relative abundance of the five most abundant phyla in diesel-spiked soil and petroleum hydrocarbon (PHC)-contaminated soil B. Wk, week

Microbial diversity profiling was conducted to identify the relative proportion of microorganisms present in a mixed microbial community. The dominant five bacterial phyla and their relative abundance in two soil samples at two different times are presented in Fig. 3. Studies have shown that bacteria capable of metabolizing or tolerating complex hydrocarbons are mostly from these phyla (Yang et al. 2014; An et al. 2013; Sherry et al. 2013). *Proteobacteria* and *Actinobacteria* were the dominant phyla in the diesel-spiked soil and field PHC-contaminated soil, respectively, at the beginning of the incubation study. After 34 weeks’ incubation, the relative abundance of *Proteobacteria* in the diesel-spiked soil reduced from 53 to 9%, but there was no significant change in relative abundance in the field PHC-contaminated soil. The relative abundance of *Actinobacteria* did not change in the field PHC-contaminated soil but reduced in the diesel-spiked soil from 22 to 17%. In the diesel-spiked soil, *Firmicutes* and *Bacteroidetes* increased from 24 to 46% and nil to 27%, respectively. Conversely, in the field PHC-contaminated soil, *Chloroflexi* increased from 3 to 13%.

**Fig. 4:**
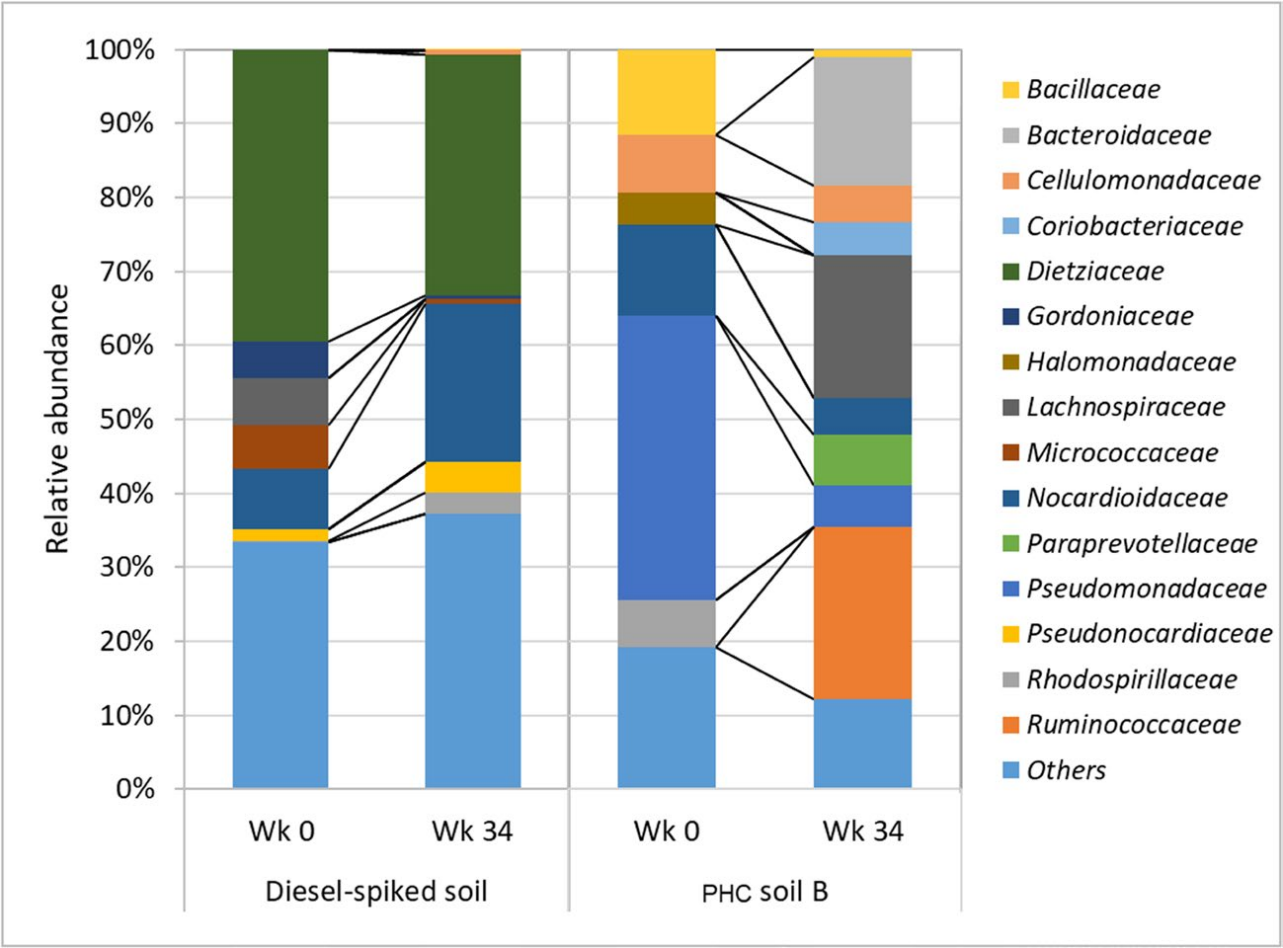
Family-level relative abundance of bacteria in diesel-spiked soil and petroleum hydrocarbon (PHC)-contaminated soil B. Wk, week

**Fig. 5:**
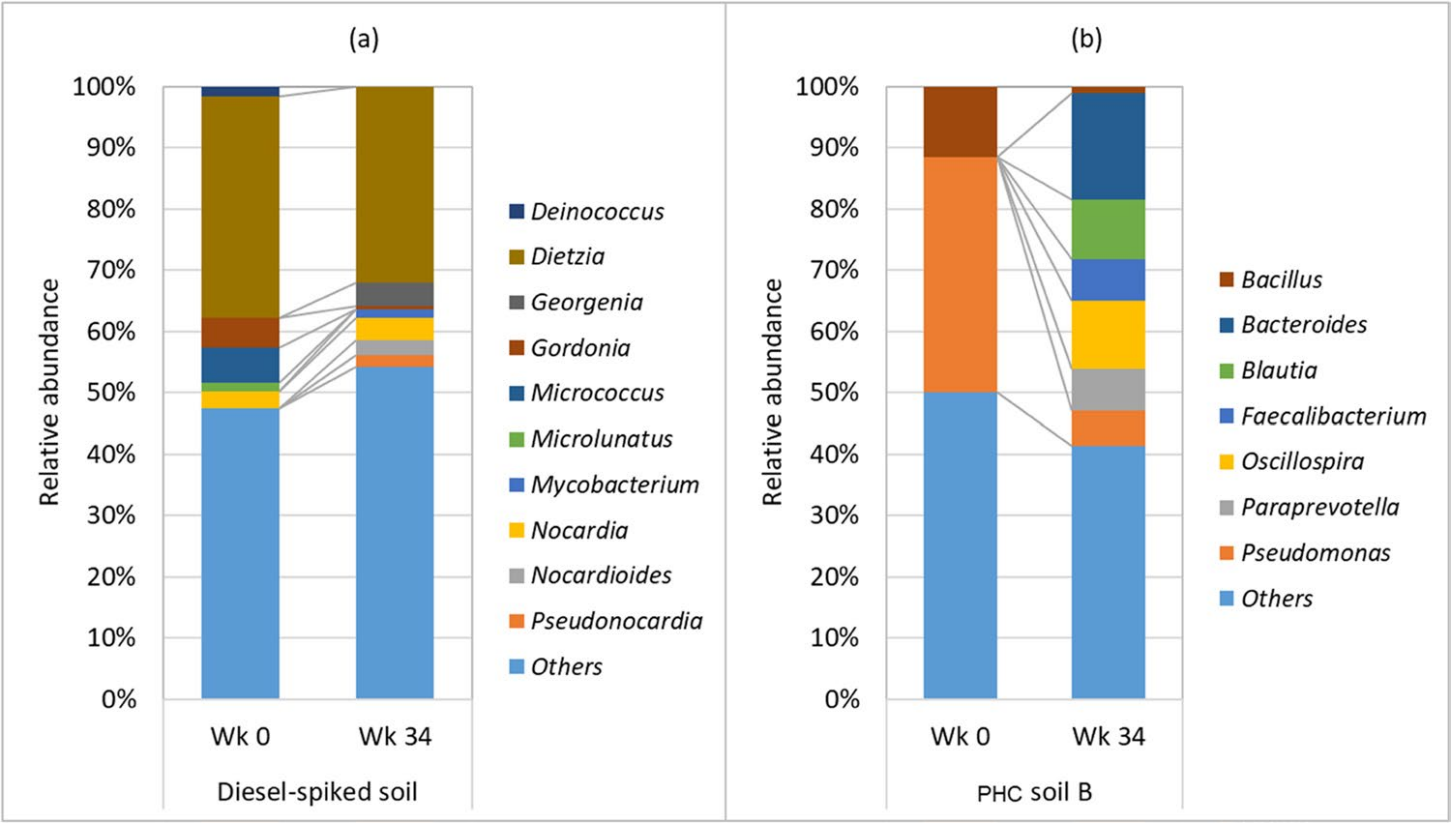
Genera-level relative abundance of bacteria diesel-spiked soil and petroleum hydrocarbon (PHC)-contaminated soil B. Wk, week

At the family level, the dominant groups in the diesel-spiked soil were *Dietziaceae* initially, which reduced from 40 to 32% after 34 weeks (Fig. 4). In the field-contaminated soil, the dominant group was *Pseudomonadaceae*, which also reduced from 38 to 5% after 34 weeks’ incubation. The families that increased in their relative abundance in the diesel-spiked soil were *Nocardioidaceae*, *Pseudomonadaceae*, and *Rhodospirillaceae*, while in the field-contaminated soils, *Bacteroidaceae*, *Coriobacteriaceae*, *Lachnospiraceae*, *Paraprevotellaceae*, and *Ruminococcaceae* increased after 34 weeks’ incubation. A significant increase of genera *Georgenia*, *Nocardia*, *Nocardioides*, and *Pseudonocardia* was observed in diesel-spiked soils after 34 weeks (Fig. 5). In contrast, in the field-contaminated soil, the genera that increased in relative abundance were *Bacteroides*, *Blautia*, *Faecalibacterium*, *Oscillospira*, and *Paraprevotella*. Substantial enrichment of these genera in the fresh PHC-contaminated soil and weathered PHC-contaminated soil with the addition of moisture to the soil suggests that they are resistant to PHC as well as utilizers of chemicals present in petroleum products. The genera *Nocardia*, *Nocardioides*, and *Pseudonocardia* have previously been reported as hydrocarbon-degrading bacteria (Jung et al. 2002; Hamamura et al. 2006; Yang et al. 2014).

Microorganisms can produce biosurfactants in response to hydrocarbon contamination. These surface-active compounds increase the surface area of hydrophobic water-insoluble substrates, which increases their bioavailability in turn, consequently promoting bacterial growth and accelerating bioremediation (Ron and Rosenberg 2002). *Nocardia* sp. have been reported as a biosurfactant producer when it was grown with hydrocarbon compound (*n*-hexadecane) as a carbon source (Kim et al. 2000). A complete genome sequencing of a *Nocardia* strain by Yang et al. (2019) showed that it contains many genes responsible not only for biosurfactant synthesis but also for emulsification and other hydrocarbon degradation-related metabolism.

A variety of indigenous microorganisms can degrade PHC compounds, and they will dominate the community following contamination or stimulation in their growth. In this study, there was a clear shift of bacterial communities in both soils after 34 weeks’ aging incubation. The relative dominance of these bacteria varied with time as well. Mainly, the abundance of *Bacteroides* and *Firmicutes* increased in diesel-spiked soil and *Chloroflexi* in weathered soil. The relative abundance of these taxa can serve as an indicator of hydrocarbon degradation in contaminated soils.

### Changes of soil properties and nutrient content

The changing pH value in the soils over the 42 weeks’ aging incubation is presented in Fig. S9. The initial pH levels in the diesel-spiked soil and PHC soils A and B were 7.85, 7.29, and 7.88, respectively. The nutrient availability is greater at this pH because of the equal number of H^+^ and OH^–^ ions. Therefore, the pH of the soils was favorable to the microbial metabolism of organic compounds. The pH in the PHC-contaminated field soils increased before 18 weeks, then decreased and remained stable after 26 weeks’ incubation. In contrast, the pH in the diesel-spiked soil increased initially, then declined after 18 weeks and rose again after 26 weeks. A change in the composition of hydrocarbon compounds can influence the chemical properties of soil. Minor fluctuations in soil pH may be primarily attributed to microbial activity instead of changes in PHC concentrations. It has been suggested that particular bacteria involved in PHC degradation produce weak acids and bases, which can lead to alterations in soil pH (Tang et al. 2012; Varjani 2017).

Historically, the primary limiting nutrients in the microbial degradation of PHCs have been understood as N and P. N is vital to cellular production, and its supplementation increases the hydrocarbon degradation effectiveness. The initial N content in the soils used for aging incubation was in the range 0.02%–0.05%, which then decreased over the incubation period (Fig. S10). Since no additional nutrients were added to the soils, the N content became very limiting after 18–26 weeks’ incubation. This might have resulted in low DHA and slow PHC degradation.

Dissolved organic matter (DOM) in the soil is produced during litter and soil organic matter decomposition and solubilization, and the DOM is generally quantified by the analysis of DOC. The initial total concentration of DOC in the diesel-spiked soil (389 mg/kg) was higher than in the PHC-contaminated weathered soils (122–177 mg/kg). DOC concentration in the diesel-spiked soil increased by 77% over the aging period and by 32% in PHC soil B after 42 weeks’ aging incubation (Fig. S11). The total concentration of DOC derived from soil is suggested here to have a positive relationship with the microbial activity in soil, which was also previously reported by Kiikkilä et al. (2014).

### PHC degradation rate constants

Rate constants help to evaluate the contribution of attenuation processes and the anticipated time required to achieve remediation goals. There are three types of rate constant that are valuable indicators that attenuation is occurring: (a) concentration versus time rate constant, (b) concentration versus distance rate constant, and (c) biodegradation rate constant (US EPA 1999). However, the application of these rate constants depends on environmental media. Unlike groundwater, the contaminants in soil are less mobile and less dispersive. Therefore, the concentration versus time rate constant could be appropriate to calculate the PHC attenuation rate in contaminated soil.

The rates of hydrocarbon degradation in the natural attenuation process were calculated using the first-order kinetic model, which has been adequately described in other studies for hydrocarbon degradation data (Nocentini et al. 2000; Namkoong et al. 2002). The results of PHC degradation rate constants and correlation for the regression analysis (*R*^2^ values) are presented in Fig. S12. The highest degradation rate constants were found in diesel-spiked soils for both TRH F2 (1.712 per year) and F3 (1.16 per year). In PHC soil A, the degradation rate of F2 was higher than that of F3 and F4.

The lowest degradation rates were estimated in the heavily weathered soil B for all TRH fractions. The *R*^2^ values of TRH F2 and F3 in diesel-spiked soils were 0.98 and 0.96, respectively, higher than the field-contaminated soils (PHC soils A and B). Diesel-contaminated soils had no TRH F4, since the carbon number of compounds in diesel went up to 20–22. TRH F2 in PHC-contaminated soils did not exhibit a good fit with exponential or linear models, but rather showed a polynomial relationship. TRH F2 in these soils decreased at the initial incubation stage (week 10), then increased at week 18 then decreased again. These fluctuations could be due to the degradation of the heavier fractions of hydrocarbons to lighter fractions, which were later degraded by the microbes. The *R*^2^ values for the curve fit of TRH F3 and F4 of soil A were 0.79 and 0.76, while in PHC soil B, the values were 0.76 and 0.54, respectively.

It is hypothesized that the degradation dynamics of hydrocarbons are influenced by their structural properties, with aliphatic hydrocarbons typically degrading more readily than aromatic ones. This differential degradation leads to the accumulation of aromatic hydrocarbons and the formation of unresolved complex mixture (Fig. 1), which can prolong the natural attenuation process. Future studies should consider these structural differences to accurately estimate degradation rates and attenuation periods.

### Projected time required for remediation goal

The projected time to achieve the cleanup goals for each representative soil type is shown in Table 5. The calculated periods varied for different TRH fractions. The NEPM ESLs are developed for individual TRH fractions; clean up should be considered complete when all fractions reach the ESLs. Therefore, the time calculated for diesel-spiked soil with a higher decay rate to achieve the NEPM ESLs for industrial land use was only 3.1 years (Table 5). The degradation rates of TRH fractions in weathered soils A and B were lower than in the diesel-spiked soil. So, the projected time to achieve the NEPM ESLs for these two soils was 4.6 and 9.5 years, respectively.

**Table 5:** Point decay rate (*k*_point_) of natural attenuation and the projected time required to reach a cleanup goal

Soil	TRH fraction (F)	NEPM ESL* (mg/kg)	TRH in soil (mg/kg)		Estimated rate and time required		Rate and time significant at 95% confidence	
			Incubation study first sample March, 2020	Incubation study last sample January, 2021	Rate (per year)	Time (years)	Rate (per year)	Time (years)
Diesel-spiked soil	F2 (> C10–C16)	170	8961	2357	1.712	2.3	1.334	3.1
	F3 (> C16–C34)	1700	14,216	5203	1.160	1.8	0.881	2.5
	F4 (> C34–C40)	3300	< 250	< 250	–	–	–	–
PHC soil A	F2 (> C10-C16)	170	490	270	0.746	1.5	2.312	0.8
	F3 (> C16–C34)	1700	12,334	8383	0.424	4.6	0.137	15.1
	F4 (> C34–C40)	3300	9110	6550	0.421	2.3	0.116	9.6
PHC soil B	F2 (> C10–C16)	170	488	457	0.292	3.4	0.458	2.8
	F3 (> C16–C34)	1700	15,173	12,300	0.233	9.5	0.051	44.6
	F4 (> C34–C40)	3300	10,361	9657	0.156	7.3	0.036	34.3

*National Environmental Protection Measures (NEPM) ecological screening levels (ESLs) are for industrial land use and coarse soils

*PHC*, petroleum hydrocarbon; *TRH*, total recoverable hydrocarbon

Since the soils are highly heterogeneous and there is natural scatter in the long-term monitoring data, there is uncertainty in estimating the natural attenuation rate and the projected timeframe to reach the cleanup goal. To deal with this uncertainty, a confidence interval was calculated for each estimate of the attenuation rate at a predetermined level of confidence. The “level of confidence” is simply the probability that the actual rate is contained within the calculated confidence interval. The nonlinear regression analysis was conducted using SPSS to calculate the degradation rate constant and time required to achieve the cleanup goal at a 95% confidence interval.

As shown in Table 5, the estimated rates of natural attenuation of TRH F3 for soil A and soil B were 0.424 and 0.233 per year, respectively, which would require 4.6 and 9.5 years, respectively, for the ESLs to be attained. At a 95% confidence level, the lower boundary of the estimated rate was 0.137 and 0.051 per year, requiring 15 and 45 years, respectively, to reach the cleanup goal. However, this does not necessarily mean that the actual time to achieve cleanup will be 45 years for soil B; it simply means that the length of time required is estimated to be no more than 45 years at a 95% level of confidence. The time required to clean up any contamination site must be determined to ascertain if its remediation can be completed in a reasonable and acceptable period, and what the estimated costs will be. The key components in determining the required time to clean up include the site-specific decay rate and the known cleanup concentration goal for the site. The time to clean up the studied soils is the projected time for different fractions of TRH to degrade to the NEPM ESLs via natural attenuation. However, recognizing the complexity of natural attenuation in arid regions, it is essential to consider the distinct environmental conditions that could influence the degradation process. Factors such as reduced moisture availability, temperature extremes, and specific soil characteristics play a critical role in determining the rate of hydrocarbon degradation. To bridge the gap between our incubation study findings and their applicability to arid landscapes, developing a calibration approach that accounts for these unique conditions is necessary. This approach might include (i) empirical models that that integrate environmental variables specific to arid regions, such as soil moisture content, temperature variability, and organic matter availability, to predict natural attenuation rates; and (ii) field validation from field studies and pilot projects in arid regions to gather empirical data, validating and refining laboratory-derived models.

## Conclusion

This study characterized weathered hydrocarbon-contaminated soils from arid regions, tested their toxicity, and assessed the aging effect for natural attenuation. The soils were found to be moderately alkaline and contain significant amounts of molybdenum, and TRH fraction analysis showed that they were moderately to heavily weathered. Ecotoxicological assessment demonstrated that hydrocarbon contamination was toxic to native plants and earthworms. Natural attenuation rate was higher in clean soil spiked with diesel compared to field-contaminated weathered soils. Soil DHAs were found to be associated with hydrocarbon degradation rates and can serve as an indicator of microbial degradation of PHCs in soil. The contrasting shift of microbial communities can also be used to indicate the biodegradation of lighter and heavier fractions of total PHCs. Additionally, the projected time required to achieve the remediation objective was calculated using degradation rate constants, which can help formulate remediation plans for contaminated sites. Although natural attenuation of PHC contamination is a low-cost and safer approach for arid soils, limited nutrients in the soil may eventually limit the biodegradation rate in the long run.

### Supplementary Information

Below is the link to the electronic supplementary material.

Supplementary file1 (DOCX 1485 KB)

## Data Availability

Data have been presented as much as possible in the manuscript. Any additional query will be available on request.
