# Supplementary material for: Residual hydrocarbons in long-term contaminated soils: implications to risk-based management
Source: Environ Sci Pollut Res Int. 2024 Feb 27;31(15):22759–73. doi: 10.1007/s11356-024-32593-7 (PMC10997687; doi:10.1007/s11356-024-32593-7)
Supplement: Supplementary file 1 — Supplementary file1 (DOCX 1485 KB) [file 11356_2024_32593_MOESM1_ESM.docx]

**Supplementary Material**

**Residual hydrocarbons in long-term contaminated soils: Implications to risk-based management**

Md Mezbaul Bahar^1,2,*^, Samarasinghe Vidane Arachchige Chamila Samarasinghe^1,2^, Dawit Bekele^1,3^, Ravi Naidu^1,2^

*^1^Global Centre for Environmental Remediation (GCER), College of Engineering, Science and Environment, University of Newcastle, Callahan NSW 2308, Australia*

*^2^crc for Contamination Assessment and Environmental Remediation (crcCARE), ATC Building, University Drive, Callahan NSW 2308, Australia*

*^3^Douglas Partners, West End QLD 4101, Australia*

**Supplementary Figures**

**
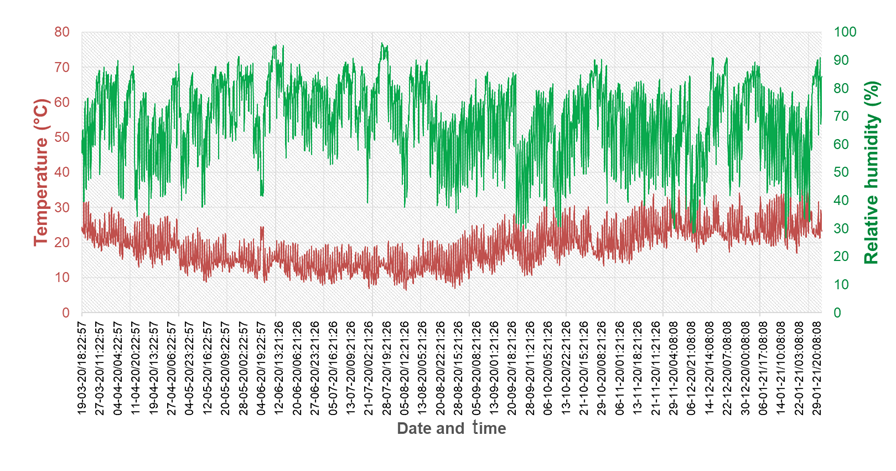
**

#### Fig. S1. Ambient conditions of aging effect incubation study. Diurnal variation of temperature and relative humidity during March 2020–January 2021.


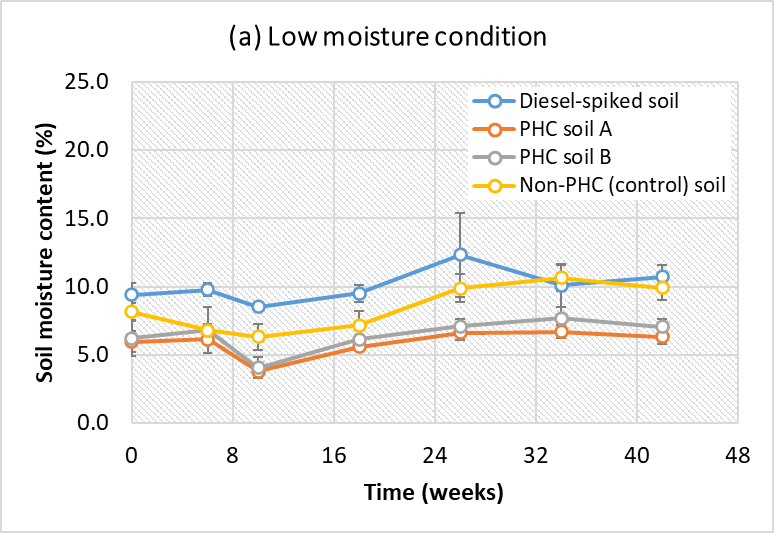

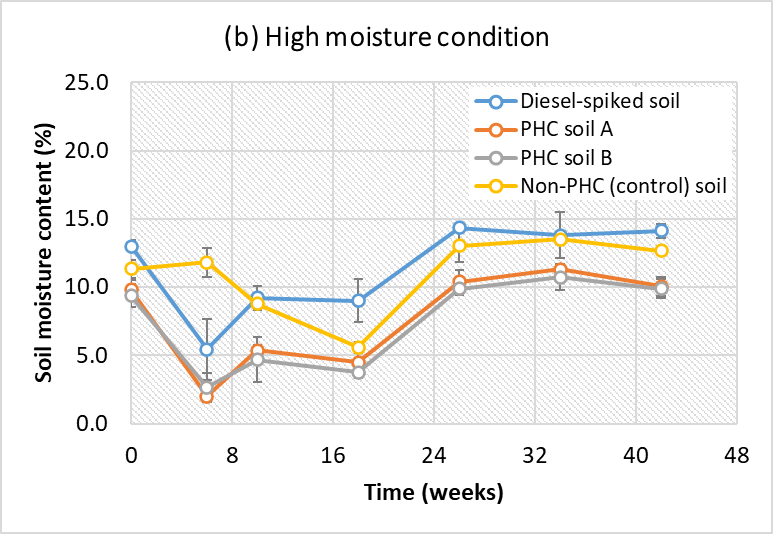


#### Fig. S2. Soil moisture content variation during aging study.

####
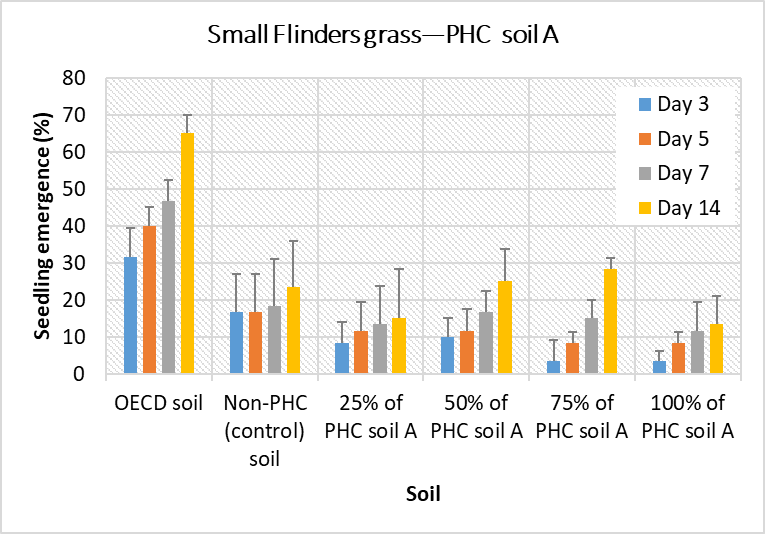

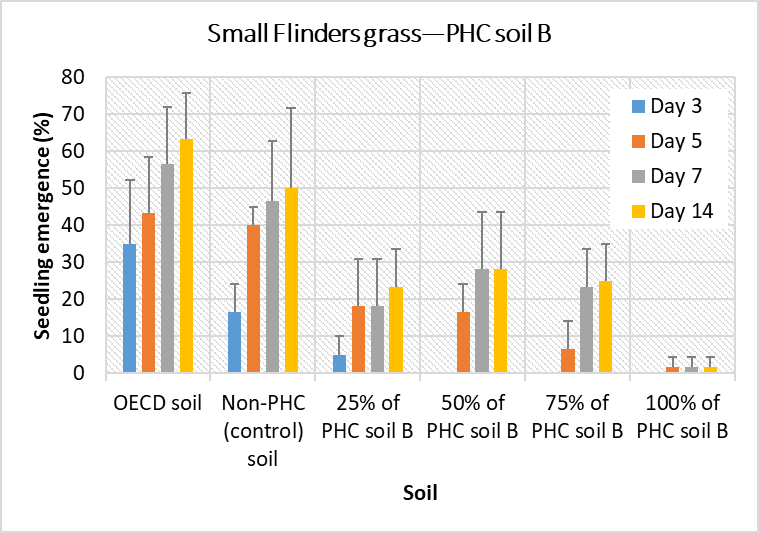


#### Fig. S3. Percentage of small Flinders Grass (*Iseilema membranaceum*) seedling emergence. Seedling emergence was tested in OECD soil, control soil, and petroleum hydrocarbon (PHC)–contaminated soil.

OECD: Organisation for Economic Co-operation and Development

####
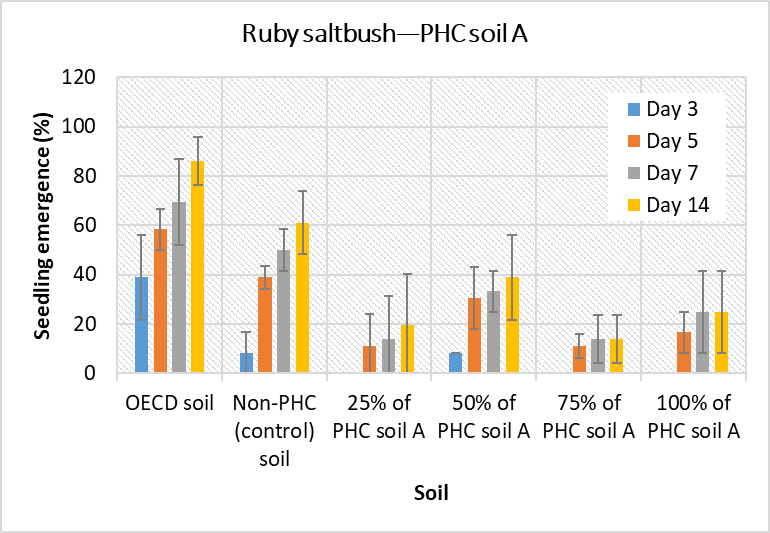

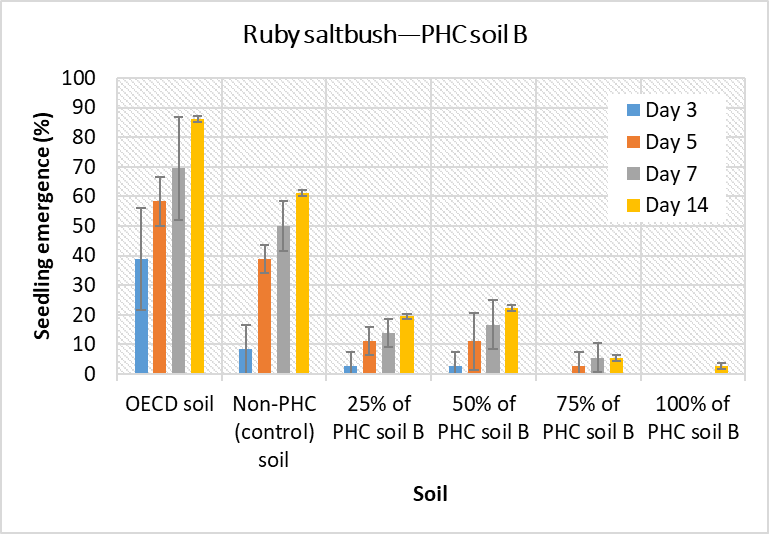


#### Fig. S4. Percentage of ruby saltbush (*Enchylaena tomentosa*) seedling emergence in OECD soil, control soil, and peat-amended (2% w/w) petroleum hydrocarbon (PHC)-contaminated soil.

OECD: Organisation for Economic Co-operation and Development


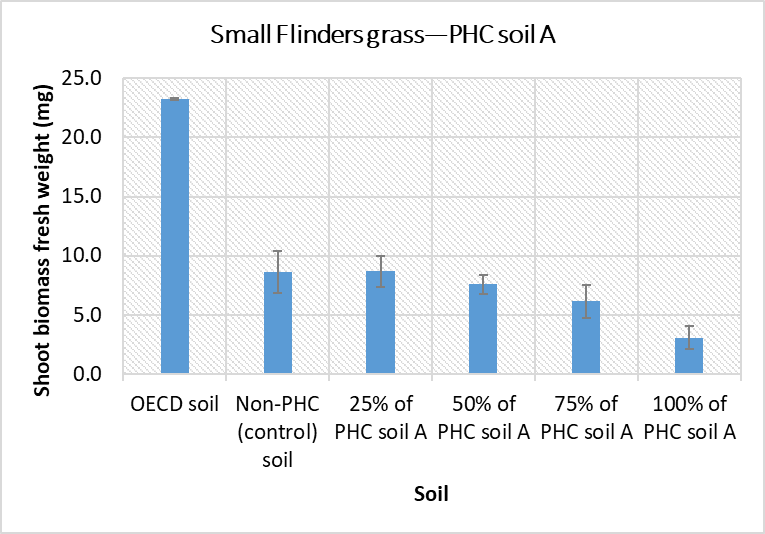

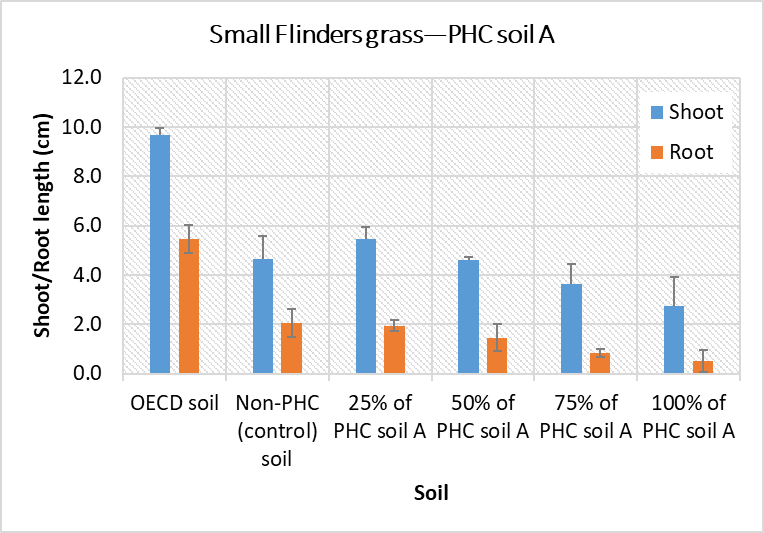

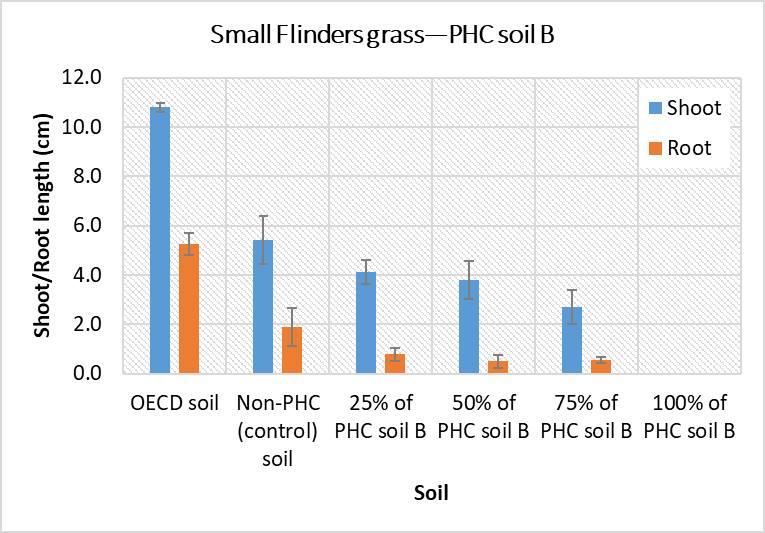

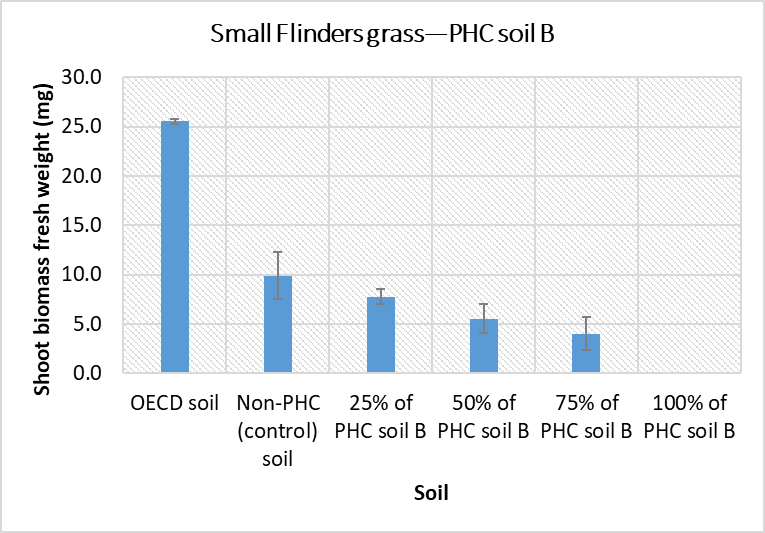


#### Fig. S5. Shoot length, root length, and shoot weight of small Flinders Grass (*Iseilema membranaceum*) in OECD soil, control soil, and PHC-contaminated soil.

#### OECD: Organisation for Economic Co-operation and Development

####
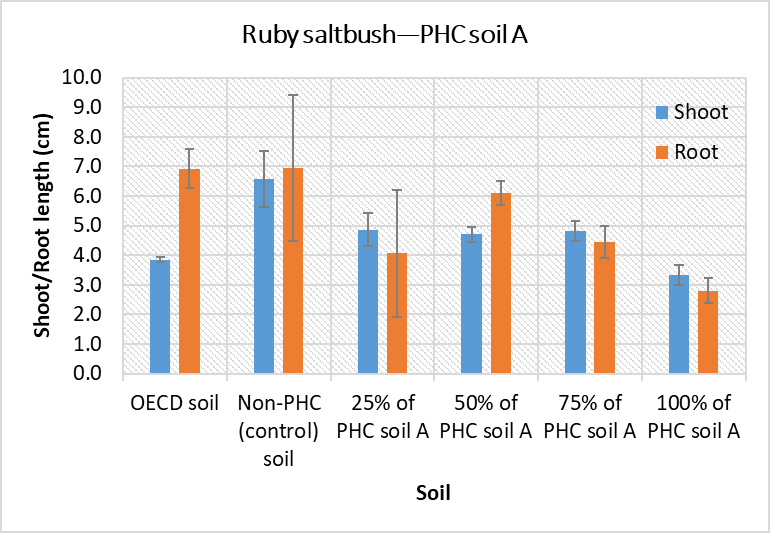

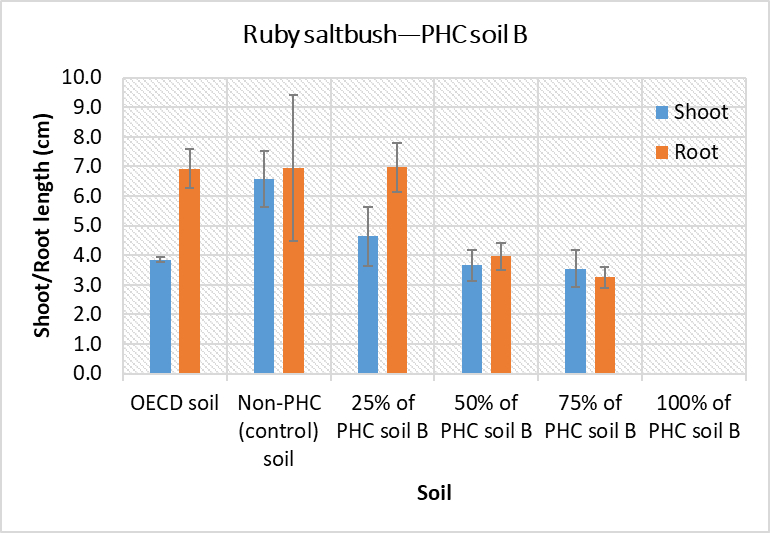


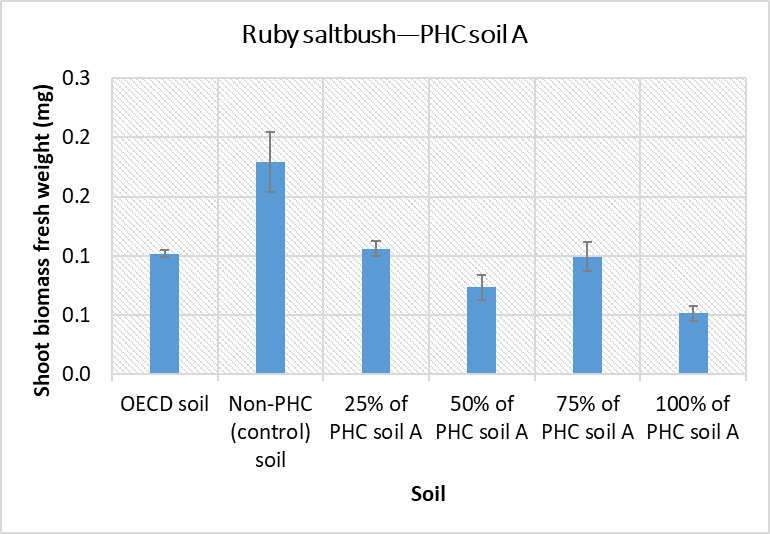

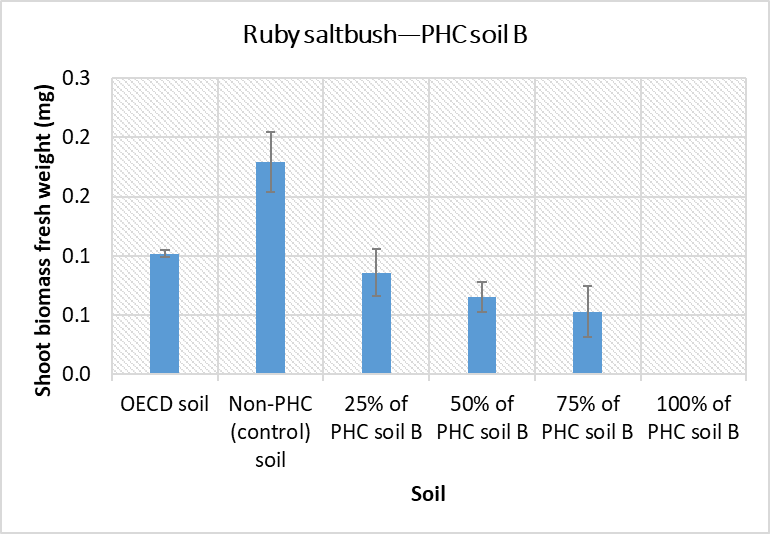


#### Fig. S6. Shoot length, root length, and shoot weight of ruby saltbush (*Enchylaena tomentosa*) in OECD soil, control soil, and peat-amended (2% w/w) PHC-contaminated soil.

#### OECD: Organisation for Economic Co-operation and Development


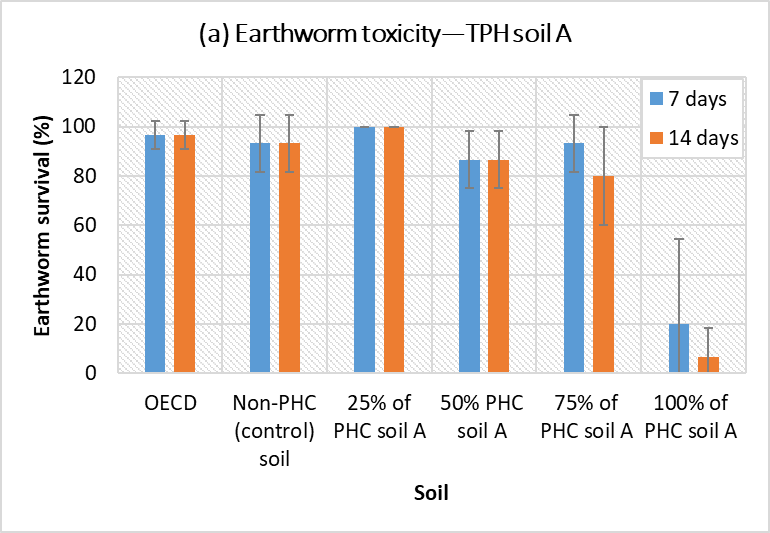

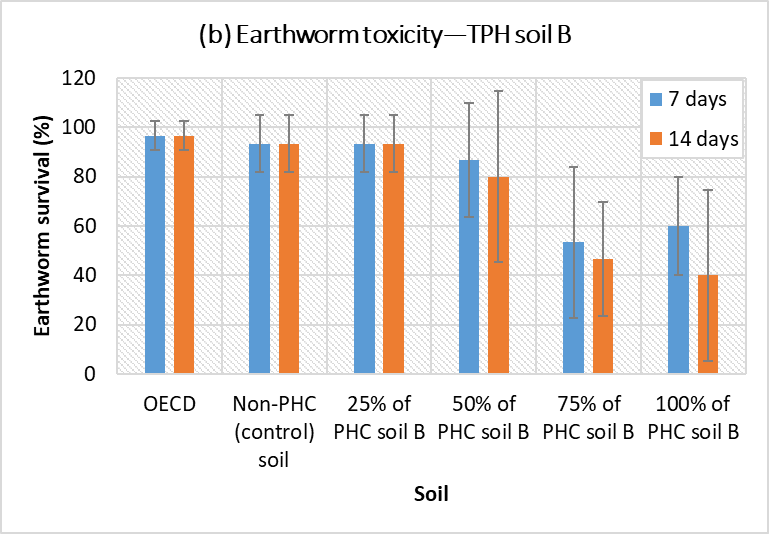


#### Fig. S7. Earthworm survival in in OECD soil, control soil, and peat-amended (2% w/w) PHC-contaminated soil.

#### OECD: Organisation for Economic Co-operation and Development (OECD)


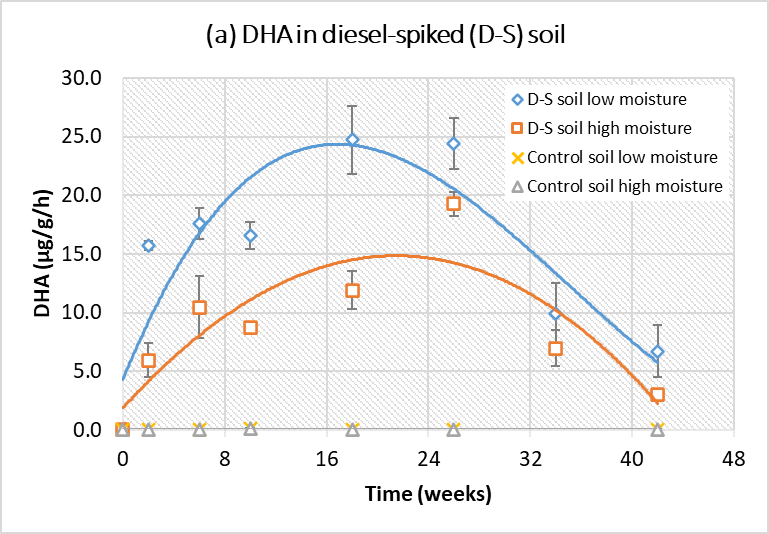


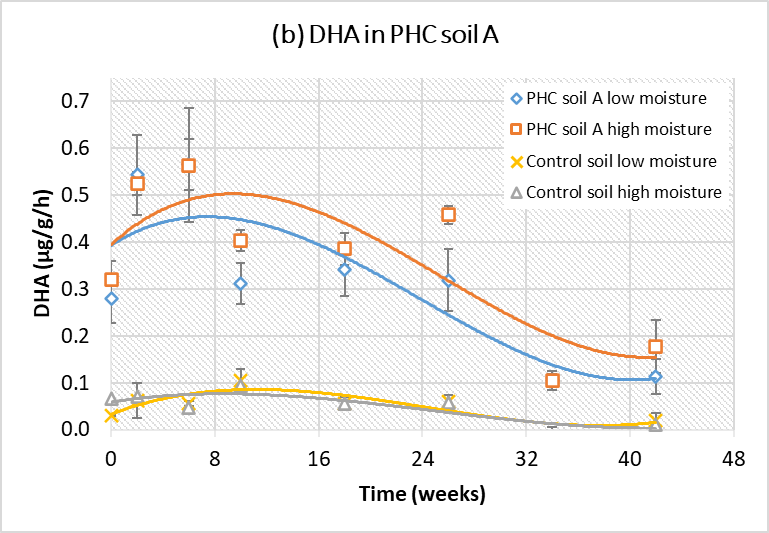


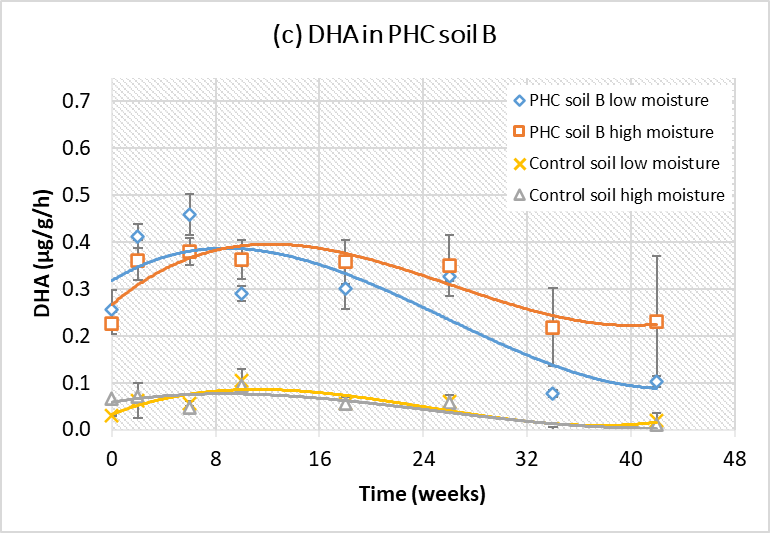


#### Fig. S8. Dehydrogenase enzyme activities (DHA) in soil. The DHA was measured in diesel-spiked soil, control soil, and petroleum hydrocarbon (PHC)–contaminated soils at two moisture conditions during the aging period.


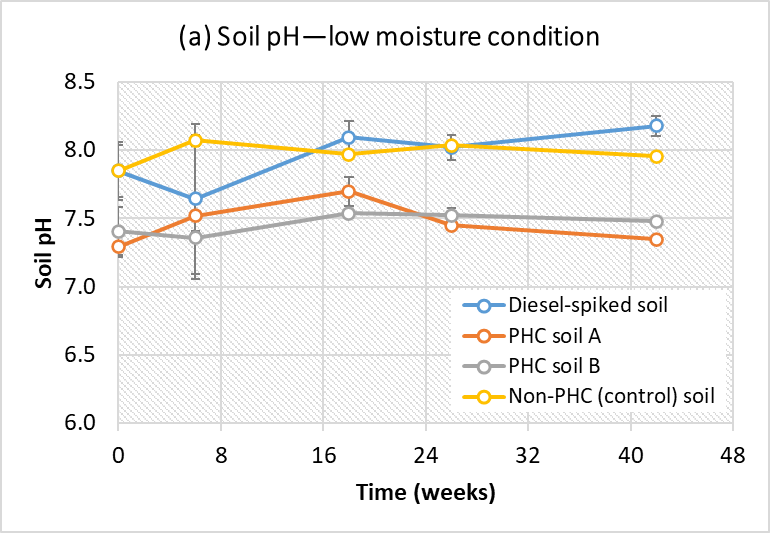

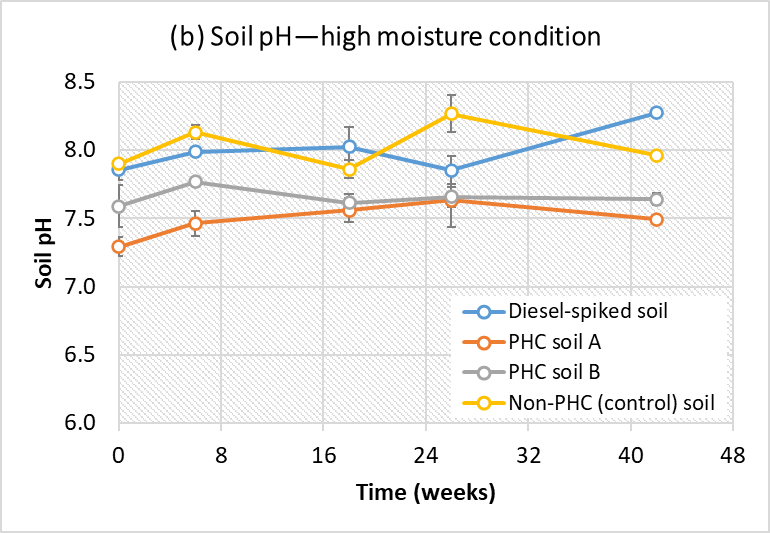


#### Fig. S9. Changes in soil pH values.


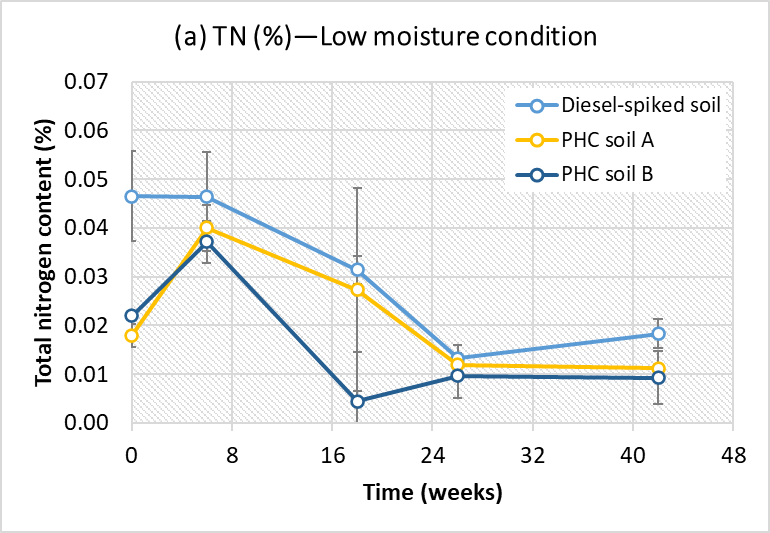

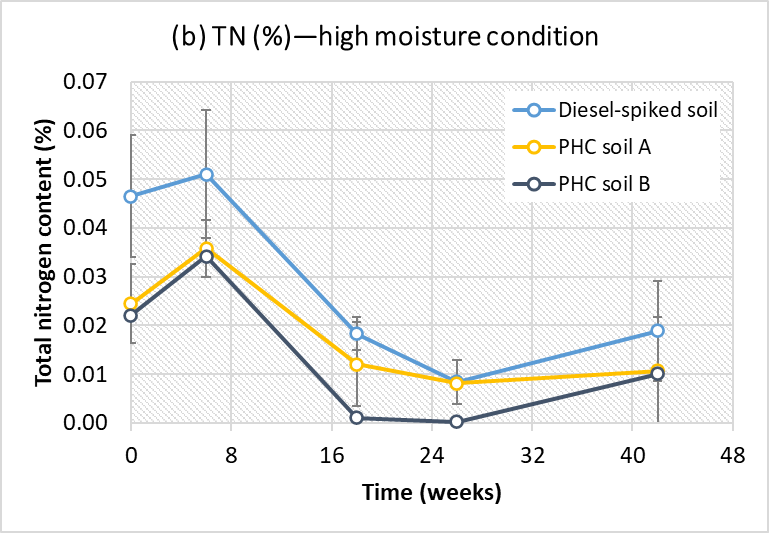


#### Fig. S10. Changes of total N content in the soils.


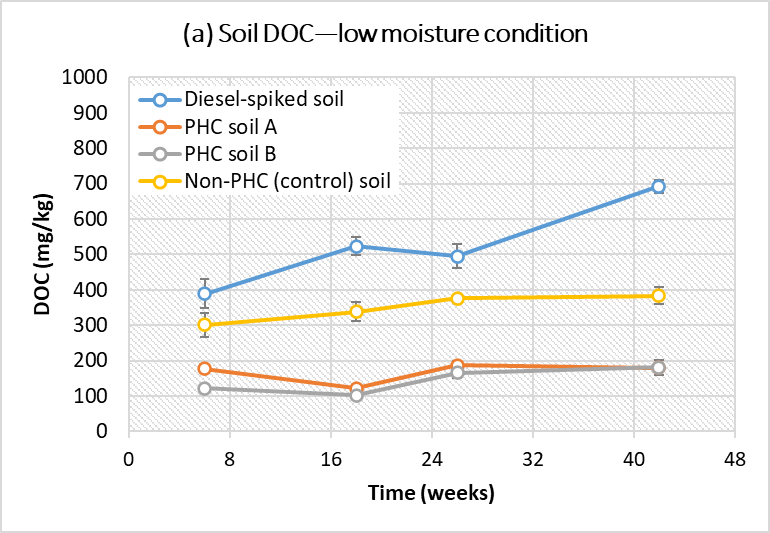

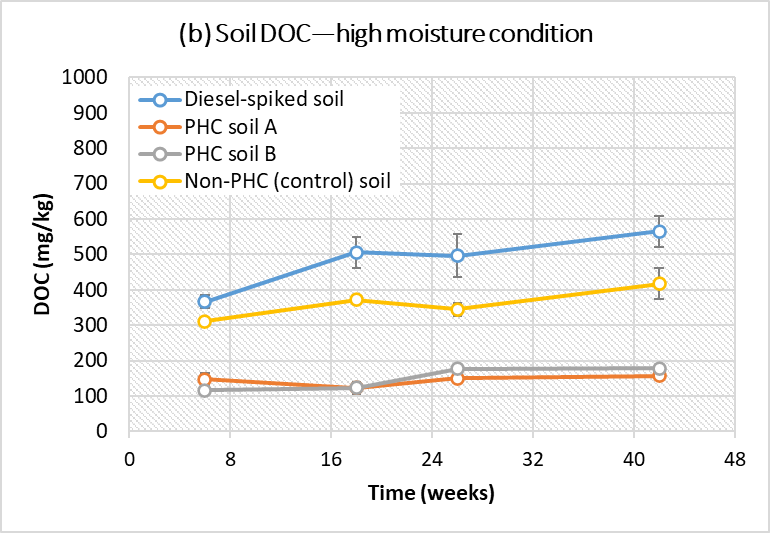


#### Fig. S11. Changes in dissolved organic carbon (DOC) concentrations in soil.

####
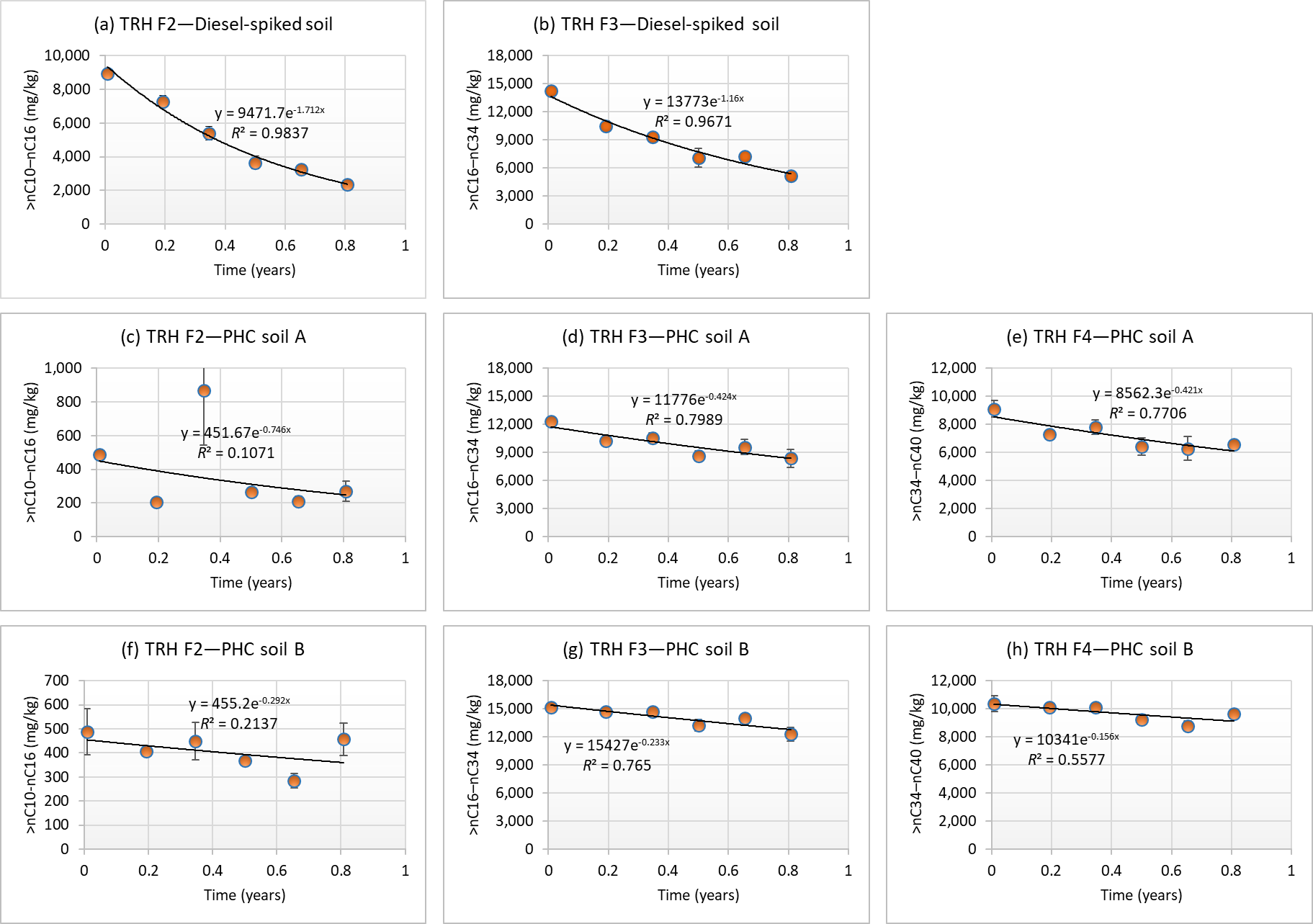


#### Fig. S12. Total recoverable hydrocarbon (TRH) degradation rates in the contaminated soils.

#### F1, F2, etc.: Fraction 1, Fraction 2, etc.
